# Association of Monogenic vs Polygenic Hypercholesterolemia With Risk of Atherosclerotic Cardiovascular Disease

**DOI:** 10.1001/jamacardio.2019.5954

**Published:** 2020-02-12

**Authors:** Mark Trinder, Gordon A. Francis, Liam R. Brunham

**Affiliations:** 1 Centre for Heart Lung Innovation, University of British Columbia, Vancouver, British Columbia, Canada; 2 Experimental Medicine Program, Department of Medicine, University of British Columbia, Vancouver, British Columbia, Canada; 3 Department of Medicine, University of British Columbia, Vancouver, British Columbia, Canada; 4 Department of Medical Genetics, University of British Columbia, Vancouver, British Columbia, Canada

## Abstract

**Importance:**

Monogenic familial hypercholesterolemia (FH) is associated with lifelong elevations in low-density lipoprotein cholesterol (LDL-C) levels and increased risk of atherosclerotic cardiovascular disease (CVD). However, many individuals with hypercholesterolemia have a polygenic rather than a monogenic cause for their condition. It is unclear if a genetic variant for hypercholesterolemia alters the risk of CVD.

**Objectives:**

To assess whether a genetic variant for hypercholesterolemia alters the risk of atherosclerotic CVD and to evaluate how this risk compares with that of nongenetic hypercholesterolemia.

**Design, Setting, and Participants:**

In this genetic-association, case-control, cohort study, individuals aged 40 to 69 years were recruited by the UK Biobank from across the United Kingdom between March 13, 2006, and October 1, 2010, and followed up until March 31, 2017. Genotyping array and exome sequencing data from the UK Biobank cohort were used to identify individuals with monogenic (*LDLR*, *APOB*, and *PCSK9*) or polygenic hypercholesterolemia (LDL-C polygenic score >95th percentile based on 223 single-nucleotide variants in the entire cohort). The data were analyzed from July 1, 2019, to December 30, 2019.

**Main Outcomes and Measures:**

The study investigated the association of genotype with the risk of coronary and carotid revascularization, myocardial infarction, ischemic stroke, and all-cause mortality among the overall study population and among participants with monogenic FH (n = 277), polygenic hypercholesterolemia (n = 2379), or hypercholesterolemia with undetermined cause (n = 2232) at comparable levels of LDL-C measured at study enrollment.

**Results:**

For the 48 741 individuals with genotyping array and exome sequencing data, the mean (SD) age was 56.6 (8.0) years, and 54.5% were female (n = 26 541 of 48 741). A monogenic FH variant for hypercholesterolemia was found in 277 individuals (0.57%, 1 in 176 individuals). Participants with monogenic FH were significantly more likely than those without monogenic FH to experience an atherosclerotic CVD event at 55 years or younger (17 of 277 [6.1%] vs 988 of 48 464 [2.0%]; *P* < .001). Compared with the general population, both monogenic and polygenic hypercholesterolemia were associated with an increased risk of CVD events. Moreover, among individuals with comparable levels of LDL-C, both monogenic (hazard ratio, 1.93; 95% CI, 1.34-2.77; *P* < .001) and polygenic hypercholesterolemia (hazard ratio, 1.26; 95% CI, 1.03-1.55; *P* = .03) were significantly associated with an increased risk of CVD events compared with the risk of such events in individuals with hypercholesterolemia without an identified genetic cause.

**Conclusions and Relevance:**

The findings of this study suggest that among individuals with hypercholesterolemia, genetic determinants of LDL-C levels may impose additional risk of CVD. Thus, understanding the possible genetic cause of hypercholesterolemia may provide important prognostic information to treat patients.

## Key Points

**Question:**

Does the risk of atherosclerotic cardiovascular disease (CVD) differ between individuals with monogenic hypercholesterolemia vs those with polygenic hypercholesterolemia?

**Findings:**

In this cohort study of 48 741 adults recruited by the UK Biobank, a monogenic cause for hypercholesterolemia was found in 277 participants (0.57%) and a polygenic cause in 2379 participants (4.9%). Both polygenic and monogenic causes of hypercholesterolemia appeared to be associated with an increased risk of CVD compared with hypercholesterolemia with an unknown genetic cause; however, monogenic hypercholesterolemia was associated with the greatest risk of CVD.

**Meaning:**

The findings of this study suggest that among individuals with comparable levels of low-density lipoprotein cholesterol, monogenic hypercholesterolemia may be associated with the greatest risk of CVD followed by polygenic hypercholesterolemia.

## Teaser

This cohort study uses the UK Biobank cohort data to compare the association of monogenic vs polygenic hypercholesterolemia with the risk of atherosclerotic cardiovascular disease among individuals with comparable levels of low-density lipoprotein cholesterol.

## Introduction

Familial hypercholesterolemia (FH) is an autosomal codominant genetic disorder with an estimated prevalence of 1 in 250 people.^1,2^ This disorder is caused by pathogenic variants in the *LDLR *(OMIM 606945), *APOB *(OMIM 107730), and *PCSK9* (OMIM 607786) genes that impair the clearance of low-density lipoproteins (LDLs) from the blood, leading to an increased risk of premature atherosclerotic cardiovascular disease (CVD).^3,4,5,6^ Despite its prevalence, FH remains underdiagnosed and undertreated.^7^

Compared with patients with elevated LDL cholesterol (LDL-C) levels and no FH-associated variant, those with a monogenic FH-associated variant have an approximately 2- to 3.5-fold increased risk of CVD.^3,6^ However, in many individuals with a phenotype of clinical FH, a monogenic FH-associated variant cannot be identified.^8,9,10,11,12,13,14^ These individuals may have a polygenic, environmental, or unknown monogenic cause for their hypercholesterolemia.

Polygenic hypercholesterolemia is estimated to account for approximately 20% to 30% of patients with clinical FH.^15,16^ The risk of CVD for individuals with polygenic hypercholesterolemia likely depends on the reference group. Genetic association and mendelian randomization studies have highlighted the important contribution of LDL-C polygenic scores to CVD risk among the general population.^17,18,19,20,21^ A recent study reported that among patients with clinical FH, an elevated LDL-C polygenic risk score was associated with increased CVD risk only in those individuals who also had monogenic FH.^22^ However, whether polygenic hypercholesterolemia vs hypercholesterolemia of unknown cause is associated with increased CVD risk remains unknown. The objective of this study was to assess how monogenic and polygenic causes of hypercholesterolemia are associated with the risk of CVD among the general population and how this risk compares with that in individuals with nongenetic hypercholesterolemia.

## Methods

### UK Biobank Cohort

**Figure 1.**
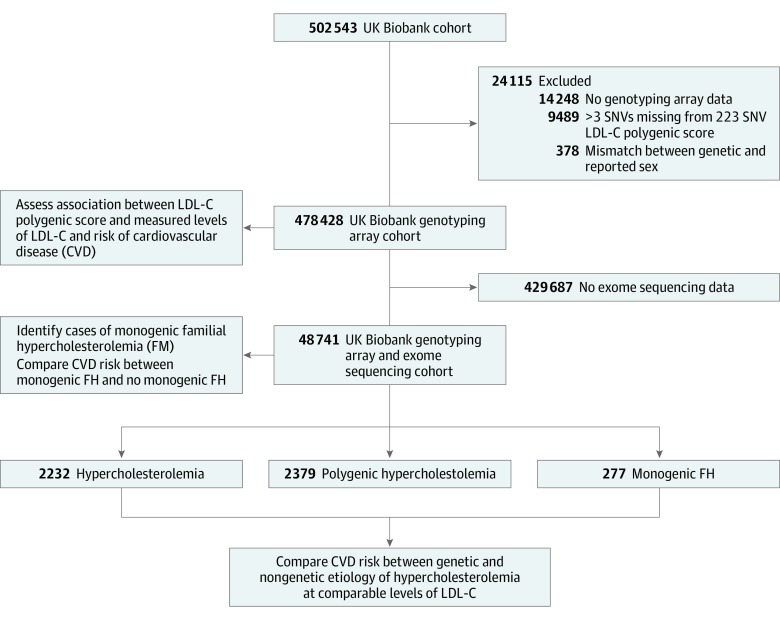
Flow Diagram of UK Biobank Cohort Subgroups Used in the Study LDL-C indicates low-density lipoprotein cholesterol; SNV, single-nucleotide variant.

This prospective cohort study included participants from the UK Biobank cohort study with genotyping array (n = 478 428) or genotyping array and exome sequencing (n = 48 741) data (Figure 1).^23,24,25^ These participants were recruited by the UK Biobank from across the United Kingdom between March 13, 2006, and October 1, 2010, and followed up until March 31, 2017. The data were analyzed from July 1, 2019, to December 30, 2019. This study was approved by the UK Biobank and the clinical research ethics board of the University of British Columbia, Vancouver, Canada. All participants provided written informed consent to participate in the UK Biobank study.

Biochemical measurements, physical examination measurements, and medical histories were assessed at the time of study enrollment unless otherwise stated (eMethods and eTable 1 in the Supplement). In patients known to be receiving cholesterol level–lowering medication at the time of enrollment, baseline LDL-C levels were estimated by multiplying LDL-C levels during treatment by 1.43, corresponding to an estimated 30% reduction in the LDL-C level.^1,26^

### Definition of CVD Events

Cardiovascular disease events were defined as coronary and carotid revascularization, myocardial infarction, ischemic stroke, and all-cause mortality. The CVD events occurring before and after enrollment were included. Events occurring prior to enrollment were identified by either self-reported medical history and/or previous hospital admission documented in an electronic health record. Incident CVD events were defined by hospital admission with an electronic health record entry only. Cardiovascular disease events were defined by diagnosis codes from the *International Classification of Diseases, Ninth Revision* and *International Classification of Diseases, Tenth Revision* (eMethods in the Supplement).^23^ Myocardial infarction, ischemic stroke, and mortality events were algorithmically defined by the UK Biobank. Coronary and carotid revascularization procedures were assessed using medical history and postenrollment operation codes according to the Office of Population Censuses and Surveys Classification of Interventions and Procedures, version 4 codes (eTable 2 in the Supplement).^23^ Events were censored on the date of loss-to-follow-up or if individuals remained event-free up to March 31, 2017.

### LDL-C Polygenic Score Construction

A data set of genotyped and imputed variants was obtained from the UK Biobank. Samples flagged for mismatch between reported sex and genetic sex or with more than 3 missing single-nucleotide variants (SNVs) of interest were excluded from analyses (Figure 1). Weighted LDL-C polygenic scores were calculated using data from the effect sizes of the 223 independent SNVs genome-wide association study discovery sample performed by the Global Lipids Genetics Consortium,^17,27^ which has been previously described (eTable 3 in the Supplement).^20^ Polygenic scores were calculated using the formula Σ [β_x_ * SNV_x_], where β_x_ is the effect size for the cholesterol-increasing allele and SNV_x_ is the number of LDL-C-increasing alleles (0, 1, or 2) for SNV_x_ (eMethods in the Supplement). K-means clustering was applied with k = 3 on the first 20 principal components of genetic ancestry to group individuals into African, East Asian, and European superpopulations (eFigure 1 in the Supplement). An individual’s LDL-C polygenic score percentile was ascertained relative to their respective UK Biobank superpopulation.

### Annotation of UK Biobank Exome Sequencing Data for Monogenic FH-Associated Variants

A population-level functionally equivalent variant data set was available from the UK Biobank in PLINK format.^25^ Variant filtering for this data set used a genotype depth filter of greater than 7 for SNVs and greater than 10 for indels and required at least 1 variant genotype to pass an allele balance (AB) filter (heterozygous SNV AB filter >0.15, heterozygous indel <0.20).

PLINK, version 1.9, was used to generate a project-level variant call file.^28^ We used SnpEff to annotate the variant call file against the GRCh38.86 reference genome and provide gene-based functional predictions.^29^ We used SnpSift to add dbNSFP^30,31^ and ClinVar^32^ annotations to variants.^33^

### Definitions of Monogenic FH, Polygenic Hypercholesterolemia, and Nongenetic Hypercholesterolemia

The *LDLR, APOB*, and *PCSK9* variants that were annotated in ClinVar as pathogenic or likely pathogenic for FH were considered monogenic FH-associated variants.^3,32^ The *LDLR* variants that were not annotated or had conflicting interpretations of pathogenicity annotations in ClinVar were considered monogenic FH-associated variants if they had a minor allele frequency of less than 0.001 and were predicted by SnpEff to result in a loss of gene function (stop gained, frameshift) or were missense variants predicted to be pathogenic by at least 5 of 6 bioinformatic tools (MetaSVM, LRT, Protein Variation Effect Analyzer, MutationTaster, Polyphen2, and Sorting Intolerant from Tolerant).^3,22,34^

Participants with a 223 SNV LDL-C polygenic score higher than the 95th percentile were defined as having polygenic hypercholesterolemia. Participants with nongenetic hypercholesterolemia were identified by a 1:1 matching of individuals with polygenic hypercholesterolemia to those from the exome sequencing cohort who did not have monogenic FH based on LDL-C level, age, sex, the first 4 principal components of genetic ancestry, and genotyping array and batch. Matching was completed using the nearest neighbor algorithm in the MatchIt package, version 3.0.2, of R statistical software (R Core Team).

### Statistical Analyses

Data were analyzed from July 1, 2019, to December 30, 2019, using R version 3.5.1, and χ^2^ tests were used for contingency analyses. For comparison of 2 groups, normally distributed data were analyzed with an unpaired, 2-tailed *t* test, and non-normally distributed data were analyzed with a Mann-Whitney *U* test. For comparison of more than 2 groups, normally distributed data were analyzed with 1-way analysis of variance (with Tukey multiple comparison post hoc tests), and non-normally distributed data were analyzed with Kruskal-Wallis tests (with Dunn multiple comparison post hoc tests).

Multivariable linear regression models were used to assess the correlation between LDL-C levels and polygenic scores and were adjusted for age, sex, the first 4 principal components of genetic ancestry, and genotyping array and batch. Time-to-event analyses were analyzed with the Survival package, version 2.43-3, for R with Peto and Peto tests. *P* values from pairwise Peto and Peto tests of more than 2 groups were adjusted using the Benjamini-Hochberg correction. Cox regression models were used and adjusted for age, sex, the first 4 principal components of ancestry, and genotyping array and batch. When explicitly indicated, Cox regression models were additionally adjusted for LDL-C levels. Statistical significance was claimed when 2-sided *P* values were < .05.

## Results

### Association of LDL-C Polygenic Score With CVD Risk

**Figure 2.**
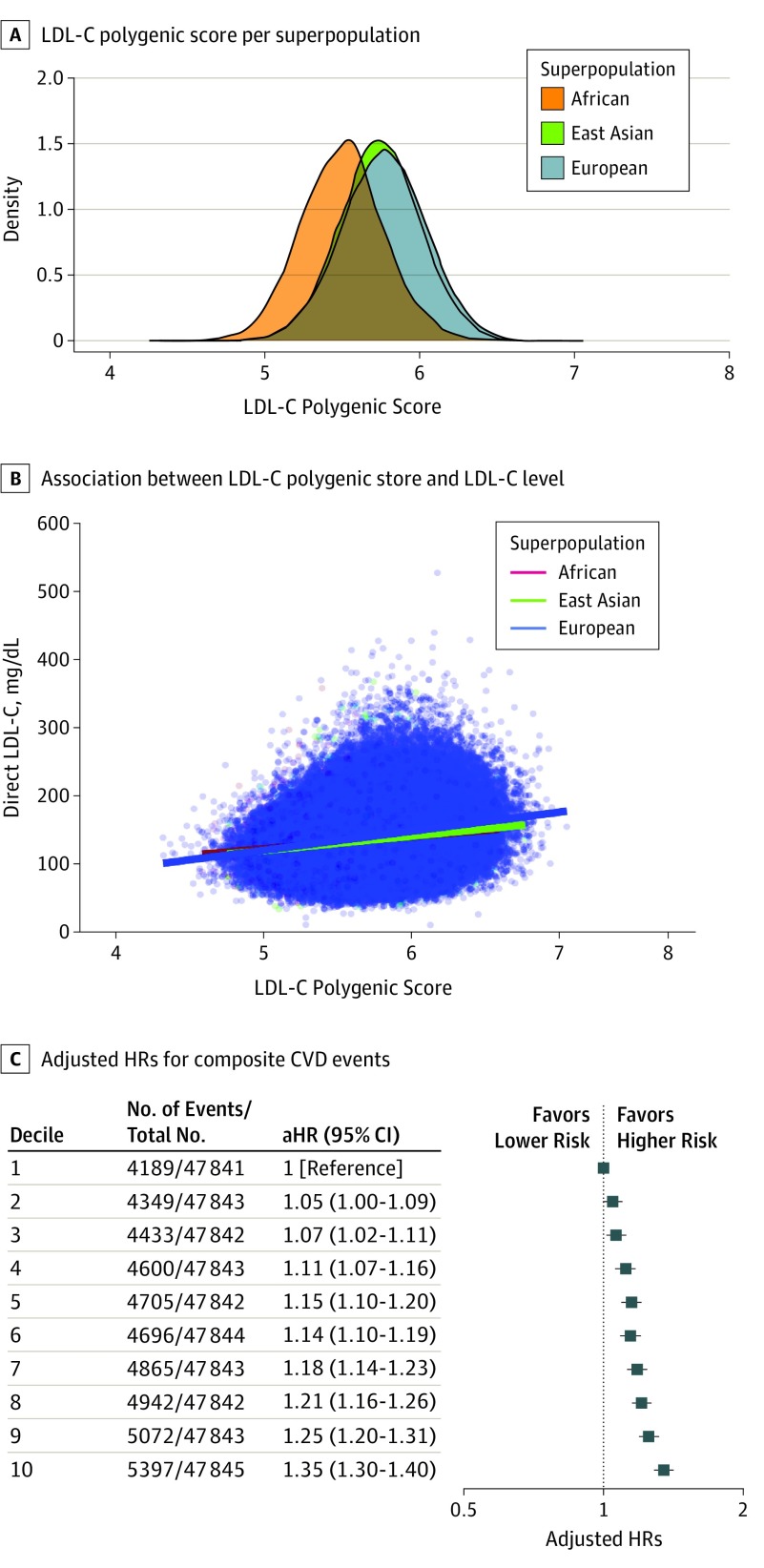
Low-Density Lipoprotein Cholesterol (LDL-C) Polygenic Scores and LDL-C Levels and Risk of Cardiovascular Disease (CVD) Among all Individuals in the Genotyping Array Cohort A, The distribution of LDL-C polygenic scores is depicted for the African, East Asian, and European ancestral superpopulations. B, The linear association between LDL-C polygenic scores and LDL-C levels (to convert to millimoles per liter, multiply by 0.0259) are depicted. Lines depict linear regression analyses segregated by ancestral superpopulation. C, The adjusted hazard ratios (aHRs) with 95% CIs for composite CVD events of myocardial infarction, coronary or carotid revascularization, ischemic stroke, or all-cause mortality are depicted for each decile of LDL-C polygenic score percentile (percentiles are calculated relative to each individual’s ancestral superpopulation). Hazard ratios were adjusted for age, sex, genotyping array and/or batch, and the first 4 principal components of ancestry.

Individuals with genotyping array data in the UK Biobank cohort study were included in the present study to assess whether LDL-C polygenic scores are associated with measured levels of LDL-C and risk of CVD (Figure 1 and eTable 4 in the Supplement). For the 478 428 individuals with genotype array data at the time of enrollment, the mean (SD) age was 56.6 (8.1) years, and 54.2% were female (n = 259 400 of 478 428). Other characteristics are provided in eTable 4 in the Supplement. There were considerable differences in the distribution of LDL-C polygenic scores among ancestral superpopulations (Figure 2A). The LDL-C polygenic scores were notably associated with baseline LDL-C levels (in milligrams per deciliter [to convert to millimoles per liter, multiply by 0.0259]) for individuals of African (multiple *R*^2^ = 0.04; β [SE] = 17.40 [1.91]; *P* < .001) (n = 4680), East Asian (multiple *R*^2^ = 0.06; β [SE] = 21.73 [1.25]; *P* < .001) (n = 10 640), and European (multiple *R*^2^ = 0.09; β [SE] = 28.01 [0.18]; *P* < .001) (n = 439 871) genetic ancestry (Figure 2B). Among the overall cohort, the correlation between LDL-C levels and LDL-C polygenic scores was comparable with the subgroup analysis restricted to individuals of European genetic ancestry (multiple *R*^2^ = 0.09; β [SE] = 27.78 [0.18]; *P* < .001) (n = 455 191).

The percentage of variance of LDL-C levels explained by the LDL-C polygenic score was assessed using stepwise addition of the 223 SNVs into multivariable linear regression models, with LDL-C levels used as the dependent variable. The percentage of variance in LDL-C levels explained by the inclusion of SNVs displayed notable saturation after 75 SNVs (9.61% variance explained) (eFigure 2 in the Supplement). An exponential plateau model of the data estimated that the maximum variance in LDL-C levels explained would be 10.19% (95% CI, 10.16-10.24; *R*^2^ = 0.97). Similar predictions were made when analyses were restricted to individuals with European genetic ancestry (eFigure 2 in the Supplement). These results suggest that incorporation of a greater number of SNVs in the LDL-C polygenic score would be unlikely to provide statistically significant improvement to the predictive ability of the LDL-C polygenic scores.

Increasing LDL-C polygenic score percentile was associated with a dose-dependent increase in CVD risk among the overall cohort (test for trend, *P* < .001) (Figure 2C). Specifically, the 10th decile of the LDL-C polygenic score percentile was associated with the greatest risk of CVD compared with the first decile of the LDL-C polygenic score (adjusted hazard ratio [aHR], 1.35; 95% CI, 1.30-1.40; *P* < .001). These results were consistent in subgroup analyses consisting of individuals of European (test for trend, *P* < .001; n = 462 236) and East Asian (test for trend, *P* = .004; n = 11 220) genetic ancestry (eFigures 3 and 4 in the Supplement). However, the cohort was underpowered to detect an association between the LDL-C polygenic score and risk of CVD events among individuals of African genetic ancestry (test for trend, *P* = .75; n = 4972) (eFigure 4 in the Supplement).

### Estimated Prevalence of Monogenic FH in the UK Biobank Cohort 

**Figure 3.**
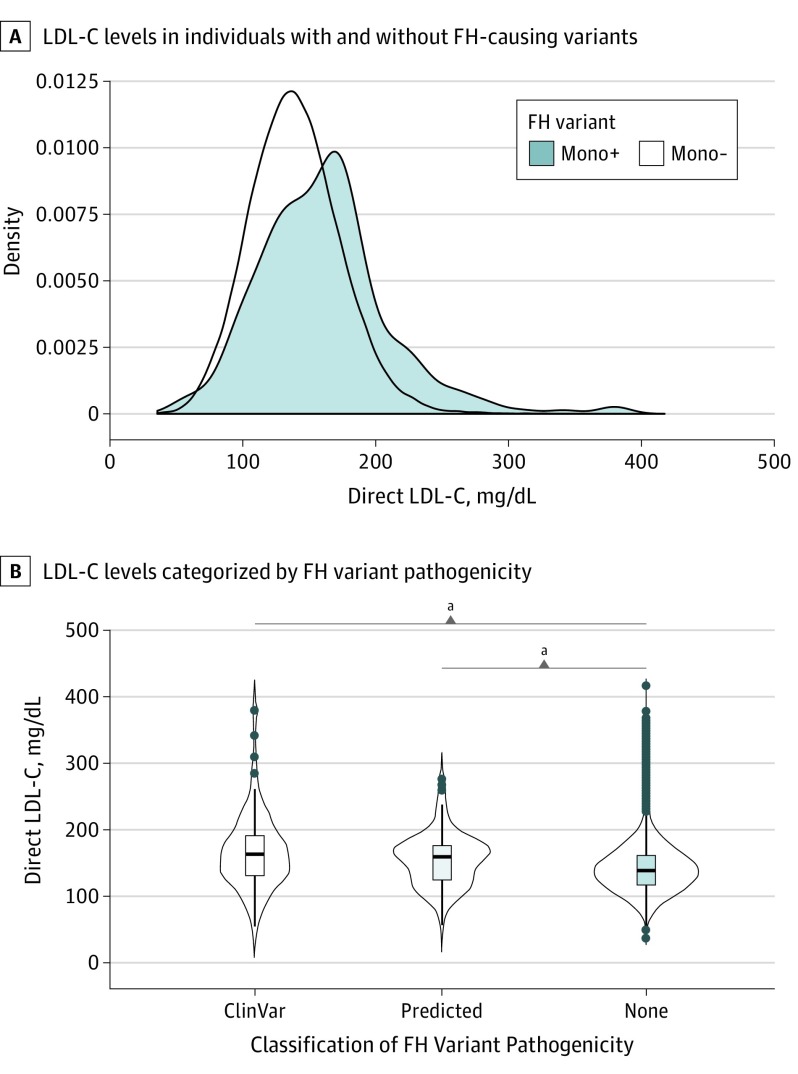
Monogenic Familial Hypercholesterolemia (FH)-Associated Variants and Elevated Low-Density Lipoprotein Cholesterol (LDL-C) Level A, Density plot shows the distribution of LDL-C levels (to convert to millimoles per liter, multiply by 0.0259) at enrollment between individuals with an FH-associated variant (mono+) and those without an FH-associated variant (mono−). B, The LDL-C levels are depicted for individuals with an FH-associated variant classified as likely pathogenic or pathogenic in ClinVar, an FH-associated variant predicted to be pathogenic, and individuals with no detected FH-associated variants. The box plots depict the median and interquartile range, with the vertical black lines displaying the minimum to maximum. ^a^*P* < .0001.

Data from the cohort of 48 741 individuals with genotyping array and exome sequencing data in the UK Biobank were used to compare polygenic and monogenic causes of FH (Figure 1 and eTables 5 and 6 in the Supplement). A predicted FH-associated variant was identified in 277 individuals, representing a prevalence of 0.57% (1 in 176 individuals). Individuals with monogenic FH had statistically significantly higher LDL-C levels than those without an FH-associated variant (mean [SD] LDL-C, 161.15 [49.1] mg/dL vs 140.2 [34.0] mg/dL; *P* < .001) (Figure 3A and eTable 6 in the Supplement). The LDL-C levels were not statistically significantly different between individuals carrying a bioinformatically predicted FH-associated variant compared with those carrying variants annotated in ClinVar as likely pathogenic or pathogenic for FH (mean [SD] LDL-C, 155.6 [39.7] mg/dL vs 167.4 [57.5] mg/dL; *P* = .38) (Figure 3B). Furthermore, the risk of premature CVD events (at 55 years or younger) was comparable in individuals carrying predicted FH-associated variants vs those carrying FH-associated variants reported in ClinVar (unadjusted HR, 3.05 [95% CI, 1.58-5.89] vs 3.05 [95% CI, 1.52-6.12]; *P* *=* .002). Pairwise comparison of Peto and Peto tests with Benjamini-Hochberg correction is shown in eFigures 5 and 6 in the Supplement.

Overall, monogenic FH-associated variants were found in *LDLR* for 257 individuals (92.9%), *PCSK9* for 13 (4.7%), and *APOB* for 7 individuals (2.5%). A total of 121 unique monogenic FH-associated variants were identified, most of which were in the *LDLR* gene (110 of 121 [90.9%]) (eTable 5 in the Supplement). None of the participants had homozygous FH; however, 2 participants carried 2 different FH-associated variants in *LDLR*.

### Monogenic FH and Premature CVD

Monogenic FH was associated with a statistically significantly greater risk of CVD vs individuals without an FH-associated variant (Peto and Peto test, χ^2^ = 9.7 on 1 *df*; *P* = .002) (eFigure 6 in the Supplement). The aHR for a composite CVD event among those with monogenic FH was 1.78 compared with that among those without a monogenic FH-associated variant (95% CI, 1.28-2.48; *P* < .001). This difference was most notable for premature CVD events (at 55 years or younger) in which there was a statistically significant enrichment for individuals with monogenic FH vs those without (17 of 277 [6.1%] vs 988 of 48 464 [2.0%]) (HR, 3.17; 95% CI, 1.96-5.12; *P* < .001).

**Figure 4.**
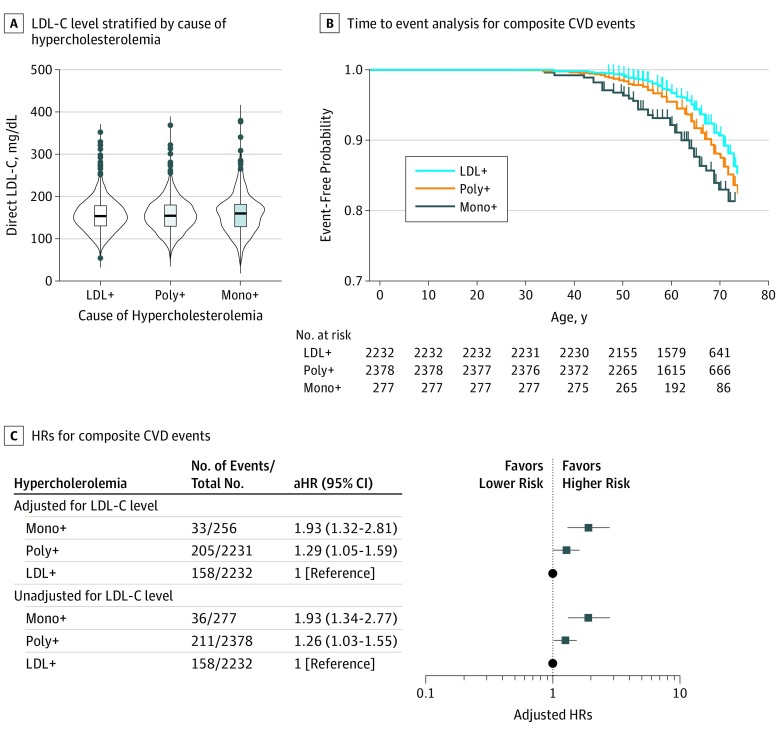
Monogenic Familial Hypercholesterolemia (FH) vs Polygenic Hypercholesterolemia and Cardiovascular Disease (CVD) Events A, Boxplots display the median, interquartile range, and minimum to maximum of direct low-density lipoprotein cholesterol (LDL-C) levels (to convert to millimoles per liter, multiply by 0.0259) that are adjusted for cholesterol-lowering medication among individuals with monogenic FH (mono+), polygenic hypercholesterolemia (poly+; 223 single-nucleotide variants LDL-C polygenic score >95th percentile), and individuals with hypercholesterolemia of no known genetic cause (LDL+). B, Time-to-first-event analyses are depicted for composite CVD events, including myocardial infarction, coronary or carotid revascularization, ischemic stroke, or all-cause mortality. Individuals were stratified based on the rank order groupings of nongenetic hypercholesterolemia, polygenic hypercholesterolemia, or monogenic FH. C, Hazard ratios (HRs) with 95% CIs are displayed for composite CVD events with or without adjustment for LDL-C levels. All HRs were adjusted for age, sex, genotyping array or batch, and the first 4 principal components of ancestry.

Next, we investigated whether there were differences in the risk of CVD events among participants with monogenic FH, polygenic hypercholesterolemia (>95th percentile of polygenic score), or nongenetic hypercholesterolemia at comparable levels of LDL-C. For this, participants were grouped based on their LDL-C levels. In this analysis, median LDL-C levels did not significantly differ among the monogenic, polygenic, and nongenetic hypercholesterolemia groups at study enrollment (Figure 4A). Characteristics of the participants stratified by these subgroups are provided in the Table. There was a significant, stepwise trend toward a greater risk of CVD among individuals with nongenetic hypercholesterolemia, polygenic hypercholesterolemia, and monogenic FH (Peto and Peto test for trend: HR, 1.93; 95% CI, 1.34-2.77; *P* < .001) (Figure 4B and C). Participants with monogenic FH had a significantly greater risk of CVD than those with polygenic hypercholesterolemia (pairwise comparison of Peto and Peto tests with Benjamini-Hochberg correction: aHR, 1.26; 95% CI, 1.03-1.55; *P* = .03) (Figure 4B and C). These results remained unaltered when HRs were also adjusted for LDL-C levels measured at study enrollment (Figure 4C). These data suggest that genetic contributions to hypercholesterolemia significantly increase CVD risk.

**Table.** Baseline Characteristics of UK Biobank Participants in the Exome Sequencing Cohort Stratified by Cause of Elevated Low-Density Lipoprotein Cholesterol (LDL-C) Levels

Characteristic	No. (%)^a^			*P* Value
	Nongenetic Hypercholesterolemia (n = 2232)	Polygenic Hypercholesterolemia (n = 2379)	Monogenic Hypercholesterolemia (n = 277)	
Age, mean (SD), y	56.3 (7.8)	56.4 (8.0)	57.1 (7.9)	.25
Sex, female	1216 (54.5)	1314 (55.2)	157 (56.7)	.74
Ancestry				
European	2153 (96.5)	2281 (95.9)	260 (93.9)	.10
East Asian	53 (2.4)	60 (2.5)	11 (3.9)	.28
African	26 (1.2)	38 (1.6)	6 (2.2)	.27
Biochemistry				
Total cholesterol, mg/dL, mean (SD)	237.33 (46.44)	236.35 (48.31)	232.91 (57.43)	.08
No. of patients	2232	2237	257	NA
Direct LDL-C, mg/dL, mean (SD)	155.36 (37.25)	155.71 (37.57)	161.15 (49.14)	.40
No. of patients	2232	2232	256	NA
Apolipoprotein B, mg/dL, mean (SD)	112.62 (25.05)	113.17 (25.73)	110.86 (26.78)	.40
No. of patients	2218	2223	253	NA
Triglycerides, mg/dL, mean (SD)	161.22 (87.15)	158.99 (96.38)	134.55 (75.22)	<.001
No. of patients	2228	2233	255	NA
HDL-C, mg/dL, mean (SD)	57.17 (14.58)	57.02 (15.04)	57.40 (14.58)	.69
No. of patients	2101	2114	244	NA
Apolipoprotein A1, mg/dL, mean (SD)	155.82 (27.05)	154.83 (27.14)	152.83 (25.8)	.24
No. of patients	2090	2103	244	NA
Lipoprotein(a), mg/dL, mean (SD)	19.91 (21.25)	18.32 (20.45)	18.86 (18.74)	.09
No. of patients	1778	1787	194	NA
Hemoglobin A_1c_, %	5.43 (0.53)	5.45 (0.60)	5.48 (0.56)	.26
No. of patients	2094	2221	257	NA
C-reactive protein, mg/dL, mean (SD)	0.25 (0.38)	0.23 (0.39)	0.25 (0.44)	<.001
No. of patients	2227	2228	255	NA
Physical examination				
BMI, mean (SD), kg/m^2^	27.60 (4.64)	27.40 (4.97)	27.59 (4.97)	.04
No. of patients	2230	2375	276	NA
Medical history, No. of patients/total No. (%)				
Angina	37/2230 (1.7)	87/2376 (3.7)	16/275 (5.8)	<.001
Myocardial infarction	29/2230 (1.30)	54/2376 (2.3)	17/275 (6.2)	<.001
Ischemic stroke	15/2230 (0.7)	34/2376 (1.5)	3/275 (0.1)	.04
Hypertension	542/2230 (24.3)	612/2376 (25.8)	73/275 (26.6)	.45
Diabetes	85/2228 (3.8)	124/2375 (5.2)	16/277 (5.8)	.05
Current smoker	208/2225 (9.4)	217/2373 (9.1)	16/277 (5.8)	.14
Medications, No. of patients/total No. (%)				
Cholesterol-lowering medication	178/1210 (14.7)	247/1307 (18.9)	57/157 (36.3)	<.001
Antihypertensives	194/1210 (16.0)	213/1307 (16.3)	34/157 (21.7)	.20
Insulin	9/1210 (0.7)	8/1307 (0.6)	0/157 (0)	.54
Exogenous hormones	110/1210 (9.1)	148/1307 (11.3)	8/157 (5.1)	.20

Abbreviations: BMI, body mass index (calculated as the weight in kilograms divided by height in meters squared); HDL-C, high-density lipoprotein cholesterol; LDL-C, low-density lipoprotein cholesterol; NA, not applicable; SNV, single-nucleotide variants.

SI conversion factors: To convert total cholesterol, LDL-C, HDL-C to millimoles per liter, multiply by 0.0259; apolipoprotein A1 and apolipoprotein B to grams per liter, multiply by 0.01; triglycerides to millimoles per liter, multiply by 0.0113; to convert lipoprotein(a) to micromoles to liter, multiply by 0.0357; and C-reactive protein to nanomoles per liter, multiply by 9.524.

^a^ Individuals were grouped on the basis of LDL-C levels. Polygenic hypercholesterolemia is defined as an LDL-C polygenic score greater than the 95th percentile within a genetic superpopulation using a 223 SNV score.

### Underdiagnosed and Undertreated Hypercholesterolemia

Individuals with monogenic FH were significantly more likely than those without a monogenic FH-associated variant to be receiving cholesterol-lowering medication at the time of enrollment (36.3% [57 of 157] vs 12.6% [3309 of 26 245]; *P* < .001). However, among individuals with monogenic FH and severe hypercholesterolemia (LDL-C level ≥193.35 mg/dL), 34.3% (12 of 35) of individuals were not receiving cholesterol-lowering medication and 40.0% (14 of 35) were seemingly unaware of their high cholesterol level at enrollment (high cholesterol level was not reported at enrollment). Undertreatment of severe hypercholesterolemia was even more notable among individuals who did not have monogenic FH, with 71.7% (1426 of 1990) of these individuals not receiving cholesterol-lowering medication at enrollment and 75.4% (1521 of 1990) unaware of their high cholesterol level.

Follow-up lipid levels were available for 128 of 1438 individuals who had severe hypercholesterolemia and were not taking cholesterol-lowering medication at study enrollment. After a mean follow-up of 3 years, only 22.7% (29 of 128) of these individuals reported taking cholesterol-lowering medication. The 99 individuals who remained untreated had a mean (SD) follow-up LDL-C level of 257.70 (22.91) mg/dL. In contrast, the mean (SD) LDL-C level of 29 individuals who had received cholesterol-lowering treatment since enrollment was 130.08 (22.76) mg/dL and all individuals had an LDL-C level of less than 193.35 mg/dL (eFigure 7 in the Supplement). These real-world data indicate the need for better recognition and management of severe hypercholesterolemia and FH.

## Discussion

The present study reports that a monogenic FH-associated variant is present in 1 in 176 (0.57%) participants from the UK Biobank exome sequencing cohort^25^ and is associated with elevated levels of LDL-C and increased risk of premature CVD. At comparable levels of LDL-C, both monogenic FH and polygenic hypercholesterolemia appeared to be associated with significantly increased risk of CVD events compared with hypercholesterolemia with no identified genetic cause, with monogenic FH associated with the greatest risk. These data suggest that the mechanism underlying hypercholesterolemia and the measured level of LDL-C contribute to CVD risk, which underscores the importance of ascertaining the causes of hypercholesterolemia to accurately assess risk.

We found that monogenic FH and polygenic hypercholesterolemia are associated with greater risk of CVD than hypercholesterolemia without a known genetic cause. Consistent with previous studies, we believe our data demonstrated that an elevated LDL-C polygenic score is associated with a moderate increase in CVD risk in both the overall population and in individuals with hypercholesterolemia.^17,18^ These findings are consistent with those of a previous report that found that monogenic FH was associated with significantly greater CVD risk than polygenic hypercholesterolemia at similar levels of LDL-C measured at study enrollment.^22^ One possible explanation for the increased risk is that monogenic hypercholesterolemia may manifest earlier in life than either polygenic hypercholesterolemia or hypercholesterolemia with no identified genetic cause, leading to greater cumulative LDL-C exposure.^3,35^ Although it is not possible to directly test this hypothesis in the UK Biobank cohort because of the absence of longitudinal lipid levels, we noted that 60.0% of individuals with monogenic hypercholesterolemia compared with 24.6% of individuals with polygenic hypercholesterolemia reported a history of high cholesterol at the time of enrollment to the UK Biobank study, which may be consistent with earlier manifestation of hypercholesterolemia in those with a monogenic cause. It is also possible that polygenic hypercholesterolemia responds better than monogenic FH to pharmacological management strategies such as cholesterol-lowering medication.

We estimated that 1 in 176 individuals from the UK Biobank cohort carried an FH-associated variant. This estimate is slightly higher than in other studies that have estimated the prevalence of FH to be in the range of 1 in 217 to 250 people.^1,2,36^ A reason for potential overestimation of the prevalence of monogenic FH is the challenge in classifying the pathogenicity of missense variants. We sought to mitigate the risk of overclassification by requiring at least 5 of 6 bioinformatic tools to predict that a missense variant was pathogenic. Furthermore, we observed that bioinformatically predicted FH-associated variants were associated with comparable LDL-C levels and similar risk of premature CVD compared with variants with likely pathogenic or pathogenic annotations in ClinVar.^37,38^ Of note, we found that many individuals with variants annotated as pathogenic for FH did not display severe hypercholesterolemia. The incomplete penetrance of FH variants we observed in the UK Biobank cohort may reflect the healthy volunteer selection bias of the participants in this cohort compared with studies of FH patients recruited from cardiology or lipid clinics.^39,40^ The results from the present study appear to highlight an important challenge to the diagnosis and treatment of individuals with monogenic FH. Future research will be required to assess how genetic background and environmental factors modulate the phenotype of monogenic FH. The finding that 2497 individuals from the exome sequencing cohort had LDL-C levels greater than 193.35 mg/dL and had neither a monogenic nor polygenic explanation for this phenotype suggests the presence of other genetic or gene-environment causes for severe hypercholesterolemia that remain to be identified.^41^

Our findings also support the possible need for routine genetic testing of patients with clinically suspected FH to reduce the incidence of premature CVD observed among individuals with monogenic FH.^3,6,42,43^ The autosomal codominant inheritance of monogenic FH-associated variants enables clinicians to efficiently screen relatives of probands in a process known as cascade screening.^44,45,46^ Cascade screening facilitates early diagnosis and initiation of lifestyle modification^47^ and cholesterol-lowering medication.^4,48,49^ Individuals with monogenic FH have the highest risk of CVD^3,50^ and are likely to derive the most benefit from statin and ezetimibe therapy, and, if needed, more costly cholesterol-lowering medications such as proprotein convertase subtilisin/kexin type 9 (PCSK9) inhibitors.^51,52,53^

### Limitations

This study has some limitations. First, data on cholesterol-lowering medication at enrollment and pretreatment lipid profiles were not available for all individuals; therefore, we used pretreatment LDL-C levels in these individuals.^1,26^ Furthermore, longitudinal data for lipid levels were not available for most participants in the UK Biobank cohort. Second, detailed characteristics needed for deep phenotyping of FH, such as thorough family history of CVD and physical examination findings suggestive of hypercholesterolemia, were not available for participants in the UK Biobank cohort. Third, the UK Biobank cohort was predominantly composed of individuals with British white genetic ancestry and individuals of non-European ancestry were underrepresented. In addition, we did not assess *LDLR* copy number and larger insertion or deletion variants, which are believed to be causative for approximately 5% of clinical FH cases.^54,55,56^ We used a polygenic risk score composed of 223 SNVs. Larger, genome-wide scores composed of millions of SNVs may provide incremental improvements in risk prediction.^21^

## Conclusions

Monogenic FH and polygenic hypercholesterolemia were associated with an increased CVD risk compared with hypercholesterolemia without an identifiable genetic cause, with monogenic FH associated with the greatest risk. These results suggest that a possible genetic cause of hypercholesterolemia is associated with CVD risk and underscores the importance of genetic profiling to better stratify risk in patients.

## Notes

Supplement. **eMethods**. **eTable 1.** Serum Biochemistry Assay Details **eTable 2.** Coding Events Used for Coronary and Carotid Revascularization Procedures **eTable 3.** Single Nucleotide Variants Used for Determining LDL-C Polygenic Scores **eTable 4.** Baseline Characteristics of UK Biobank Participants at the Time of Study Enrollment **eTable 5.** FH-causing Variants Identified in the LDLR, APOB, and PCSK9 Genes. **eTable 6.** Baseline Characteristics of UK Biobank Participants in the Exome Sequencing Cohort Stratified by Monogenic FH **eFigure 1.** Classification of Patients by Genetic Ancestry Superpopulation. **eFigure 2.** Percent Variance in LDL-C Levels Explained by Single-Nucleotide Variants (SNVs) in the LDL-C Polygenic Score **eFigure 3.** Measured Levels of LDL-C Versus Decile of LDL-C Polygenic Score Percentile **eFigure 4.** LDL-C Polygenic Scores are Associated With Risk of CVD Among the East Asian and European Superpopulations **eFigure 5.** Impact of ClinVar Pathogenic Versus Predicted FH-causing Variants on Premature CVD Events **eFigure 6.** Impact of Monogenic FH-causing Variants on CVD Events **eFigure 7.** Hypercholesterolemia is Underdiagnosed and Undertreated **eReferences.**
