# Supplementary material for: Association of Monogenic vs Polygenic Hypercholesterolemia With Risk of Atherosclerotic Cardiovascular Disease
Source: JAMA Cardiol. 2020 Feb 12;5(4):390–9. doi: 10.1001/jamacardio.2019.5954 (PMC7042820; doi:10.1001/jamacardio.2019.5954)
Supplement: Supplement. — eMethods. eTable 1. Serum Biochemistry Assay Details eTable 2. Coding Events Used for Coronary and Carotid Revascularization Procedures eTable 3. Single Nucleotide Variants Used for Determining LDL-C Polygenic Scores eTable 4. Baseline Characteristics of UK Biobank Participants at the Time of Study Enrollment eTable 5. FH-causing Variants Identified in the LDLR, APOB, and PCSK9 Genes. eTable 6. Baseline Characteristics of UK Biobank Participants in the Exome Sequencing Cohort Stratified by Monogenic FH eFigure 1. Classification of Patients by Genetic Ancestry Superpopulation. eFigure 2. Percent Variance in LDL-C Levels Explained by Single-Nucleotide Variants (SNVs) in the LDL-C Polygenic Score eFigure 3. Measured Levels of LDL-C Versus Decile of LDL-C Polygenic Score Percentile eFigure 4. LDL-C Polygenic Scores are Associated With Risk of CVD Among the East Asian and European Superpopulations eFigure 5. Impact of ClinVar Pathogenic Versus Predicted FH-causing Variants on Premature CVD Events eFigure 6. Impact of Monogenic FH-causing Variants on CVD Events eFigure 7. Hypercholesterolemia is Underdiagnosed and Undertreated eReferences. [file jamacardiol-5-390-s001.pdf]

Supplementary Online Content

Trinder M, Francis GA, Brunham LR. Association of monogenic vs polygenic hypercholesterolemia with risk of atherosclerotic cardiovascular disease. *JAMA Cardiol.* Published online February 12, 2020. doi:10.1001/jamacardio.2019.5954

eMethods.
eTable 1. Serum Biochemistry Assay Details
eTable 2. Coding Events Used for Coronary and Carotid Revascularization Procedures
eTable 3. Single Nucleotide Variants Used for Determining LDL-C Polygenic Scores
eTable 4. Baseline Characteristics of UK Biobank Participants at the Time of Study Enrollment
eTable 5. FH-causing Variants Identified in the LDLR, APOB, and PCSK9 Genes
eTable 6. Baseline Characteristics of UK Biobank Participants in the Exome Sequencing Cohort Stratified by Monogenic FH
eFigure 1. Classification of Patients by Genetic Ancestry Superpopulation
eFigure 2. Percent Variance in LDL-C Levels Explained by Single-Nucleotide Variants (SNVs) in the LDL-C Polygenic Score
eFigure 3. Measured Levels of LDL-C Versus Decile of LDL-C Polygenic Score Percentile
eFigure 4. LDL-C Polygenic Scores are Associated With Risk of CVD Among the East Asian and European Superpopulations
eFigure 5. Impact of ClinVar Pathogenic Versus Predicted FH-causing Variants on Premature CVD Events
eFigure 6. Impact of Monogenic FH-causing Variants on CVD Events
eFigure 7. Hypercholesterolemia is Underdiagnosed and Undertreated
eReferences.

This supplementary material has been provided by the authors to give readers additional information about their work.

© 2020 Trinder M et al. *JAMA Cardiology.*

eMethods

LDL-C polygenic score.

The percent variance of LDL-C levels explained by the LDL-C polygenic as assessed by stepwise addition of the 223 SNVs into multivariable linear regression models with LDL-C levels as the dependent variable. The linear regression models were adjusted for age, sex, the first 4 principal components of genetic ancestry, and genotyping array and batch. SNVs were added in the order displayed in Online-Only Table 2 (from largest-to-smallest absolute effect size. The percent variance in LDL-C explained versus number of SNVs in the model were assessed using an exponential plateau model with Prism 8 software (GraphPad Software, Inc.)

Serum biochemistry analyses.

The detailed description of the serum biochemistry analyses have been described (http://biobank.ndph.ox.ac.uk/showcase/showcase/docs/serum_biochemistry.pdf and http://biobank.ndph.ox.ac.uk/showcase/showcase/docs/serum_hb1ac.pdf). All serum biochemistry results described were performed using the Beckman Coulter AU5800 platform (Beckman Coulter (UK), Ltd) other than hemoglobin A1c, which was performed using Bio-Rad Variant II Turbo analysers (Bio-Rad Laboratories, Inc.). The general details for each assay are displayed in Online Only Table 1. Results for serum biochemistry are reported for samples obtained at the time of study enrolment unless otherwise stated.

Definition of CVD events.

A detailed description of the UK Biobank's algorithm for identifying an individual's first myocardial infarction or stroke event have been described (myocardial infarction: http://biobank.ndph.ox.ac.uk/showcase/showcase/docs/alg_outcome_mi.pdf; stroke: http://biobank.ndph.ox.ac.uk/showcase/showcase/docs/alg_outcome_stroke.pdf).

© 2020 Trinder M et al. *JAMA Cardiology.*

ONLINE-ONLY TABLES

eTable 1. Serum Biochemistry Assay Details

Serum assay	Assay Manufacturer	Analytical Platform	Analysis Methodology	Reported Units	Manufacturer's Analytical Range for Reported Units	Measurement Conversion
Total cholesterol (mg/dL)	Beckman Coulter (UK), Ltd	Beckman Coulter AU5800	Enzymatic	mmol/L	0.5 - 18	x 38.67
Direct LDL-C (mg/dL)	Beckman Coulter (UK), Ltd	Beckman Coulter AU5800	Enzymatic Selective Protection	mmol/L	0.26 -10.3	x 38.67
Apolipoprotein B (mg/dL)	Beckman Coulter (UK), Ltd	Beckman Coulter AU5800	Immuno-turbidimetric	g/L	0.4 - 2	x 100
Triglycerides (mg/dL)	Beckman Coulter (UK), Ltd	Beckman Coulter AU5800	Enzymatic	mmol/L	0.1 - 11.3	x 88.57
HDL-C (mg/dL)	Beckman Coulter (UK), Ltd	Beckman Coulter AU5800	Enzyme Immuno-inhibition	mmol/L	0.05 - 4.65	x 38.67
Apolipoprotein A1 (mg/dL)	Beckman Coulter (UK), Ltd	Beckman Coulter AU5800	Immuno-turbidimetric	g/L	0.4 - 2.5	x 100
Lipoprotein(a) (mg/dL)	Randox Bioscience, UK	Beckman Coulter AU5800	Immuno-turbidimetric	nmol/L	5.76 - 189	/ 2.4
Hemoglobin A1c (%)	Bio-Rad Laboratories, Inc.	Bio-Rad Variant II Turbo analysers	High Performance Liquid Chromatography	mmol/mol	15 - 184	(x 0.09148) + 2.152
C-reactive protein (mg/dL)	Beckman Coulter (UK), Ltd	Beckman Coulter AU5800	Immuno-turbidimetric	mg/L	0.08 - 80	/ 10

The measurement conversion describes the calculation used to convert reported units into the units described for the serum assay results. HDL-C: high-density lipoprotein cholesterol; LDL-C: low-density lipoprotein cholesterol.

© 2020 Trinder M et al. *JAMA Cardiology.*

eTable 2. Coding Events Used for Coronary and Carotid Revascularization Procedures

**Coronary and Carotid Revascularization Procedures**	**Coding - Definition**
Baseline medical history	• 1069 – Heart surgery<br>• 1070 – Coronary angioplasty<br>• 1095 – Coronary artery bypass grafts (CABG)<br>• 1105 – Carotid artery surgery/endarterectomy
Post-enrollment procedures during hospitalization (Office of Population Censuses and Surveys Classification of Interventions and Procedures, version 4 [OPCS-4])	• K40 – Saphenous vein graft replacement of coronary artery<br>• K41 – Other autograft replacement of coronary artery<br>• K42 – Allograft replacement of coronary artery<br>• K43 – Prosthetic replacement of coronary artery<br>• K44 – Other replacement of coronary artery<br>• K45 – Connection of thoracic artery to coronary artery<br>• K46 – Other bypass of coronary artery<br>• K49 – Transluminal balloon angioplasty of coronary artery<br>• K50 – Other therapeutic transluminal operations on coronary artery<br>• K55.3 – Open removal of cardiac thrombus<br>• K75 – Percutaneous transluminal balloon angioplasty and insertion of stent into coronary artery<br>• K76 – Transluminal operations on cardiac conduit<br>• L29 – Reconstruction of carotid artery<br>• L30.3 – Open embolectomy of carotid artery<br>• L31.1 – Percutaneous transluminal angioplasty of carotid artery<br>• L31.3 – Endovascular repair of carotid<br>• L31.4 – Percutaneous transluminal insertion of stent into carotid artery<br>• L31.8 – Other specified transluminal operations on carotid artery<br>• L31.9 – Unspecified transluminal operations on carotid artery

© 2020 Trinder M et al. *JAMA Cardiology.*

eTable 3. Single Nucleotide Variants Used for Determining LDL-C Polygenic Scores

Locus	Chr:Pos	rsID	Initial Description	Annotation	EA/NEA	EAF	Beta
LDLR	19:11202306	rs6511720	GLGC2013	Intron	T/G	0.1075	-0.211427
NA	19:45422946	rs4420638	GLGC2013	Intergenic	G/A	0.1797	0.16801
CELSR2	1:109818306	rs629301	GLGC2013	Utr3	T/G	0.7742	0.157518
NA	20:17845921	rs2328223	GLGC2013	Intergenic	C/A	0.2244	0.140
APOB	2:21263900	rs1367117	GLGC2013	Nonsynonymous	A/G	0.2844	0.105
APOH	17:64210580	rs1801689	GLGC2013	Nonsynonymous	C/A	0.02673	0.104
SUGP1	19:19407718	rs10401969	GLGC2013	Intron	C/T	0.08456	-0.089559
ABCG8	2:44072576	rs4299376	GLGC2013	Intron	T/G	0.7127	-0.07030
HPR	16:72108093	rs2000999	GLGC2013	Intron	A/G	0.2003	0.06302
NA	8:9183358	rs9987289	GLGC2013	Intergenic	G/A	0.8993	0.056878
NA	2:118835841	rs10490626	GLGC2013	Intergenic	A/G	0.06783	-0.053409
FADS1	11:61569830	rs174546	GLGC2013	Utr3	T/C	0.3121	-0.05333
NA	8:126490972	rs2954029	GLGC2013	Intergenic	T/A	0.4468	-0.0476
SLC22A1	6:160578860	rs1564348	GLGC2013	Intron	C/T	0.1517	0.047474
NA	1:55504650	rs2479409	GLGC2013	Intergenic	A/G	0.6582	-0.046614
DOCK7	1:63025942	rs2131925	GLGC2013	Intron	T/G	0.6477	0.044479
HFE	6:26093141	rs1800562	GLGC2013	Nonsynonymous	A/G	0.0479	-0.044136
ST3GAL4	11:126243952	rs11220462	GLGC2013	Intron	A/G	0.1453	0.042734
NA	7:25991826	rs4722551	GLGC2013	Intergenic	C/T	0.1577	0.039910
TIMD4	5:156390297	rs6882076	GLGC2013	Upstream	C/T	0.626	0.039039
NA	11:116648917	rs964184	GLGC2013	Intergenic	C/G	0.8478	-0.03699
VLDLR	9:2640759	rs3780181	GLGC2013	Intron	G/A	0.07446	-0.036964
HNF1A	12:121416650	rs1169288	GLGC2013	Nonsynonymous	C/A	0.3325	0.03660
TOP1	20:39672618	rs6029526	GLGC2013	Intron	A/T	0.5095	0.035212
HLA-DRA	6:32412435	rs3177928	GLGC2013	Utr3	A/G	0.1409	0.0349
NA	6:16127407	rs3757354	GLGC2013	Intergenic	T/C	0.24403	-0.03343
CMTM6	3:32533010	rs7640978	GLGC2013	Intron	T/C	0.09258	-0.0331
DNAH11	7:21607352	rs12670798	GLGC2013	Intron	C/T	0.2471	0.032799
NA	16:56993324	rs3764261	GLGC2013	Intergenic	A/C	0.3127	-0.03244

© 2020 Trinder M

NA	8:55421614	rs10102164	GLGC2013	Intergenic	A/G	0.202	0.0309521	0.003265	2.58E-21
NA	1:27138393	rs12748152	GLGC2013	Intergenic	T/C	0.07209	0.0306381	0.005127	2.29E-09
GPAM	10:113933886	rs2255141	GLGC2013	Intron	G/A	0.7281	-0.02792	0.002982	7.68E-21
NA	8:59388565	rs2081687	GLGC2013	Intergenic	C/T	0.6624	-0.0276358	0.002789	3.76E-23
TMEM57	1:25775733	rs12027135	GLGC2013	Intron	T/A	0.5089	0.0272454	0.002663	1.44E-24
NA	12:112072424	rs11065987	GLGC2013	Intergenic	G/A	0.3804	-0.02598	0.002932	8.01E-19
PLEC	8:145043543	rs11136341	GLGC2013	Intron	G/A	0.3768	0.0256472	0.003484	1.83E-13
MTMR3	22:30378703	rs5763662	GLGC2013	Intron	T/C	0.03132	0.0254959	0.008161	1.78E-03
ANXA9	1:150958836	rs267733	GLGC2013	Nonsynonymous	G/A	0.1366	-0.0253057	0.003861	5.59E-11
NYNRIN	14:24883887	rs8017377	GLGC2013	Nonsynonymous	A/G	0.4228	0.02263	0.002705	6.03E-17
BRCA2	13:32953388	rs4942486	GLGC2013	Intron	C/T	0.5158	-0.0215512	0.002653	4.48E-16
NA	17:7091650	rs314253	GLGC2013	Intergenic	C/T	0.3507	-0.01959	0.002753	1.10E-12
NA	20:12962718	rs364585	GLGC2013	Intergenic	G/A	0.6361	0.0187598	0.00284	3.99E-11
DNAJC13	3:132163200	rs17404153	GLGC2013	Intron	T/G	0.1261	-0.0182777	0.004074	7.26E-06
PPARA	22:46627603	rs4253772	GLGC2013	Intron	T/C	0.09758	0.018203	0.004683	1.01E-04
CSNK1G3	5:122855416	rs4530754	GLGC2013	Intron	A/G	0.5536	0.0169497	0.002771	9.50E-10
NA	2:121309488	rs2030746	GLGC2013	Intergenic	T/C	0.4127	0.01389	0.002665	1.88E-07
DOK7	4:3473139	rs6831256	GLGC2013	Intron	G/A	0.4412	0.01266	0.002679	2.28E-06
JAK2	9:5073770	rs77375493	MVP	Nonsynonymous	T/G	0.001137	-0.2952	0.048864	1.54E-09
ZNF574	19:42584958	rs201596848	MVP	Nonsynonymous	T/C	0.001363	-0.2553608	0.036965	4.91E-12
HIST1H1B	6:27835293	rs201148465	MVP	Synonymous	C/A	0.001378	0.2098	0.037634	2.49E-08
FGB	4:155489608	rs6054	MVP	Nonsynonymous	T/C	0.003804	0.0910444	0.021773	2.90E-05
ZFPM1	16:88580796	rs147032017	MVP	Synonymous	T/C	0.006487	-0.0906348	0.016925	8.56E-08
SERPINA1	14:94844947	rs28929474	MVP	Nonsynonymous	T/C	0.01536	0.08124	0.010758	4.30E-14
NA	9:136155000	rs635634	MVP	Intergenic	T/C	0.1904	0.0773341	0.003486	4.88E-109
NA	6:39246856	rs2758873	MVP	Intergenic	A/G	0.237758	0.0738191	0.03972	6.31E-02
HMGCR	5:74655726	rs3846663	MVP	Intron	T/C	0.3977	0.0656162	0.002748	5.11E-126
RAB21	12:72179446	rs61754230	MVP	Nonsynonymous	T/C	0.01507	0.0573498	0.010816	1.14E-07
A1CF	10:52573772	rs41274050	MVP	Nonsynonymous	T/C	0.007148	0.0570591	0.015688	2.76E-04
HNF4A	20:43042364	rs1800961	MVP	Nonsynonymous	T/C	0.03085	-0.05428	0.007587	8.45E-13
NA	7:150529449	rs17173637	MVP	Intergenic	C/T	0.1449	0.0457748	0.046854	3.29E-01

© 2020 Trinder M et al. *JAMA Cardiology.*

PXK	3:58381287	rs13315871	MVP	Intron	A/G	0.08472	-0.0383928	0.004888	4.02E-15
SLC39A8	4:103188709	rs13107325	MVP	Nonsynonymous	T/C	0.05057	-0.0345177	0.006053	1.18E-08
EVI5	1:93009438	rs7515577	MVP	Intron	A/C	0.8166	0.0302476	0.003455	2.05E-18
FUT2	19:49206417	rs492602	MVP	Synonymous	G/A	0.4502	0.028327	0.002831	1.45E-23
RPS6	9:19376255	rs67710536	MVP	Utr3	C/A	0.1111	0.0282728	0.004458	2.27E-10
CD300LG	17:41926126	rs72836561	MVP	Nonsynonymous	T/C	0.02753	-0.02729	0.008014	6.62E-04
KPNB1	17:45732774	rs11871606	MVP	Intron	A/C	0.5019	-0.0271453	0.002649	1.22E-24
MOSC1	1:220970028	rs2642438	MVP	Nonsynonymous	G/A	0.7619	0.02622	0.00793	9.46E-04
FAM117B	2:203532304	rs11694172	MVP	Intron	G/A	0.1548	-0.0254615	0.045714	5.78E-01
NA	6:135411228	rs9376090	MVP	Intergenic	C/T	0.2377	-0.0251724	0.003155	1.48E-15
SPTY2D1	11:18632984	rs10128711	MVP	Intron	C/T	0.6911	0.02502	0.003254	1.48E-14
UNK	17:73782191	rs2125345	MVP	Intron	C/T	0.3126	-0.0242084	0.002982	4.74E-16
ABCA1	9:107664301	rs1883025	MVP	Intron	T/C	0.2623	-0.02412	0.002991	7.24E-16
C20orf173	20:34116282	rs7261862	MVP	Nonsynonymous	C/T	0.1768	-0.023574	0.003492	1.47E-11
GCKR	2:27730940	rs1260326	MVP	Nonsynonymous	C/T	0.6287	-0.02315	0.002778	7.76E-17
TMPRSS11E	4:69343287	rs976002	MVP	Nonsynonymous	G/A	0.2259	0.022859	0.003272	2.84E-12
PHC1	12:9082581	rs4883201	MVP	Intron	G/A	0.115	-0.02204	0.004161	1.18E-07
NA	8:18272881	rs1495741	MVP	Intergenic	A/G	0.7527	-0.021635	0.003089	2.48E-12
VTN	17:26694861	rs704	MVP	Nonsynonymous	A/G	0.4864	0.0214349	0.002647	5.58E-16
RAF1	3:12628920	rs2290159	MVP	Intron	C/G	0.204	-0.0212431	0.003893	4.87E-08
ABCB11	2:169830155	rs2287623	MVP	Intron	A/G	0.5921	-0.0208912	0.002699	1.00E-14
TBC1D8	2:101627925	rs1062062	MVP	Nonsynonymous	T/C	0.1234	-0.02069	0.004216	9.26E-07
FAM24B	10:124610027	rs1891110	MVP	Nonsynonymous	A/G	0.5487	0.0205575	0.002645	7.69E-15
PDXDC1	16:15129940	rs3198697	MVP	Synonymous	T/C	0.3988	-0.02055	0.034098	5.47E-01
NA	18:47160953	rs7241918	MVP	Intergenic	T/G	0.8481	0.0204613	0.003704	3.32E-08
NA	5:131744574	rs1016988	MVP	Intergenic	C/T	0.2223	-0.019644	0.003195	7.81E-10
NA	14:71096344	rs9646133	MVP	Intergenic	T/G	0.3263	-0.0189208	0.002831	2.35E-11
NF1	17:29629326	rs11080150	MVP	Intron	G/A	0.3257	-0.0188852	0.002822	2.19E-11
SLC25A17	22:41170063	rs2076674	MVP	Intron	C/T	0.3538	0.0183521	0.002824	8.08E-11
TSPYL6	2:54482553	rs17189743	MVP	Nonsynonymous	A/G	0.02832	0.01822	0.007927	2.15E-02
NA	6:52453220	rs2239619	MVP	Intergenic	A/C	0.619	0.0180313	0.002769	7.42E-11

© 2020 Trinder M et al. *JAMA Cardiology.*

C7orf50	7:1083777	rs1997243	MVP	Intron	G/A	0.1426	0.01803	0.00378	1.85E-06
FGFR4	5:176520243	rs351855	MVP	Nonsynonymous	A/G	0.292	-0.01803	0.003282	3.94E-08
PNPLA3	22:44324727	rs738409	MVP	Nonsynonymous	G/C	0.2313	-0.01796	0.003135	1.00E-08
SEC16A	9:139368953	rs3812594	MVP	Nonsynonymous	A/G	0.2375	-0.0178008	0.003146	1.54E-08
CCDC109A	10:74637326	rs7901016	MVP	Intron	C/T	0.06978	-0.0177426	0.005719	1.92E-03
C14orf43	14:74250126	rs13379043	MVP	Intron	C/T	0.3053	-0.0177218	0.002961	2.16E-09
NA	6:34546560	rs2814982	MVP	Intergenic	T/C	0.1196	-0.0167865	0.004097	4.19E-05
JMJD1C	10:65027610	rs10761731	MVP	Intron	T/A	0.4199	0.01674	0.002687	4.63E-10
PGS1	17:76403984	rs4129767	MVP	Intron	A/G	0.4879	0.0165082	0.002641	4.06E-10
NA	10:94839642	rs2068888	MVP	Intergenic	A/G	0.4701	-0.0163534	0.002673	9.47E-10
NA	6:31265490	rs2247056	MVP	Intergenic	C/T	0.765	0.016079	0.003538	5.49E-06
DOCK6	19:11347493	rs737337	MVP	Synonymous	C/T	0.1197	-0.015796	0.004326	2.61E-04
PNLIPRP2	10:118397971	rs10885997	MVP	Nonsynonymous	G/A	0.4093	0.0154428	0.002888	8.91E-08
TOM1	22:35711098	rs138777	MVP	Intron	G/A	0.6172	-0.01544	0.002878	8.19E-08
ZGPAT	20:62349586	rs4809330	MVP	Intron	G/A	0.7005	-0.0152932	0.002919	1.61E-07
ASAP3	1:23766233	rs1077514	MVP	Intron	T/C	0.8219	0.0149723	0.003536	2.29E-05
TRPS1	8:116599199	rs2293889	MVP	Intron	G/T	0.6204	-0.0149623	0.00275	5.29E-08
EFNA1	1:155106227	rs4745	MVP	Nonsynonymous	T/A	0.4876	-0.0148197	0.00272	5.11E-08
MPV17L2	19:18304700	rs874628	MVP	Nonsynonymous	G/A	0.2609	0.0145906	0.00302	1.36E-06
CYP4V2	4:187120211	rs13146272	MVP	Nonsynonymous	A/C	0.6231	-0.01453	0.002723	9.50E-08
NA	11:122522375	rs7941030	MVP	Intergenic	C/T	0.38	0.01445	0.002705	9.20E-08
C6orf97	6:151901409	rs4870044	MVP	Intron	T/C	0.3408	0.0143991	0.002982	1.38E-06
TCEA2	20:62695931	rs6062343	MVP	Intron	A/G	0.4322	-0.0143458	0.002737	1.59E-07
ARHGEF15	17:8216468	rs871841	MVP	Nonsynonymous	C/T	0.5191	0.0140764	0.002749	3.05E-07
NA	7:72982874	rs17145738	MVP	Intergenic	T/C	0.1149	0.0138548	0.00418	9.18E-04
SNX13	7:17911038	rs10282707	MVP	Intron	T/C	0.4261	-0.0136602	0.003201	1.97E-05
NA	9:16887366	rs3927680	MVP	Intergenic	A/T	0.5178	-0.0131538	0.002747	1.68E-06
NA	12:57809456	rs1106766	MVP	Intergenic	T/C	0.2121	-0.01311	0.003249	5.48E-05
TMED6	16:69385641	rs76116020	MVP	Nonsynonymous	G/A	0.03279	-0.0130922	0.007433	7.82E-02
NA	1:182168885	rs1689800	MVP	Intergenic	G/A	0.3391	0.0129118	0.002784	3.53E-06
NA	8:19844222	rs12678919	MVP	Intergenic	G/A	0.09741	0.0123	0.004464	5.87E-03

© 2020 Trinder M et al. *JAMA Cardiology.*

PROX2	14:75322794	rs8014204	MVP	Intron	A/G	0.5702	0.0121151	0.002813	1.66E-05
NA	8:61548494	rs626913	MVP	Intergenic	C/A	0.5191	0.01188	0.002634	6.45E-06
NA	2:113841030	rs6734238	MVP	Intergenic	G/A	0.3909	-0.01183	0.002722	1.39E-05
NA	10:17260290	rs10904908	MVP	Intergenic	G/A	0.4456	0.011618	0.002658	1.24E-05
MARCH8	10:46013277	rs970548	MVP	Intron	C/A	0.2421	0.0113847	0.003087	2.26E-04
NA	14:64235556	rs7157785	MVP	Intergenic	T/G	0.1773	0.01136	0.003761	2.53E-03
PCIF1	20:44576502	rs7679	MVP	Utr3	C/T	0.1685	0.0110928	0.003539	1.72E-03
GAS6	13:114527838	rs7400722	MVP	Intron	A/G	0.4203	0.0108984	0.003121	4.79E-04
NA	3:135926622	rs645040	MVP	Intergenic	T/G	0.7818	0.0106915	0.003199	8.31E-04
SBNO1	12:123796238	rs4759375	MVP	Intron	T/C	0.1087	0.0103686	0.005479	5.84E-02
STX4	16:31048079	rs10871454	MVP	Intron	T/C	0.369	-0.0100139	0.002924	6.16E-04
ADCY5	3:123065778	rs11708067	MVP	Intron	G/A	0.2068	-0.0097741	0.003322	3.26E-03
ERLIN1	10:101912064	rs2862954	MVP	Nonsynonymous	C/T	0.405	0.0096841	0.002789	5.16E-04
NA	1:219687432	rs2785990	MVP	Intergenic	T/C	0.6852	0.0095037	0.002856	8.77E-04
PHLDB1	11:118486067	rs11603023	MVP	Intron	C/T	0.5788	-0.009488	0.002676	3.92E-04
SERPINF2	17:1648502	rs2070863	MVP	Nonsynonymous	T/C	0.2128	0.0094815	0.003355	4.72E-03
NA	5:55861786	rs9686661	MVP	Intergenic	T/C	0.1869	0.0094768	0.003374	4.98E-03
CECR5	22:17625915	rs35665085	MVP	Nonsynonymous	A/G	0.04979	0.0091775	0.006052	1.29E-01
DFNB31	9:117166246	rs2274159	MVP	Nonsynonymous	G/A	0.48	0.0090078	0.002666	7.30E-04
NSD1	5:176637576	rs28932178	MVP	Nonsynonymous	C/T	0.1679	-0.008877	0.003608	1.39E-02
SLC22A18	11:2936952	rs16928809	MVP	Intron	A/G	0.08745	-0.008583	0.004698	6.77E-02
AFF1	4:88030261	rs442177	MVP	Intron	T/G	0.5731	0.008547	0.002674	1.39E-03
ANKS3	16:4755108	rs78074706	MVP	Nonsynonymous	A/G	0.02152	0.008417	0.009031	3.51E-01
NA	6:43757896	rs998584	MVP	Intergenic	A/C	0.4824	0.008065	0.002752	3.39E-03
FCGR2A	1:161479745	rs1801274	MVP	Nonsynonymous	G/A	0.4842	-0.0079908	0.003603	2.66E-02
DAGLB	7:6449272	rs702485	MVP	Utr3	G/A	0.4633	0.0078124	0.033105	8.13E-01
NA	11:10388782	rs2923084	MVP	Intergenic	G/A	0.2006	0.007712	0.003356	2.16E-02
PCNXL3	11:65391317	rs12801636	MVP	Intron	A/G	0.2339	0.007643	0.003119	1.43E-02
ANGPTL4	19:8433196	rs7255436	MVP	Intron	A/C	0.5207	0.0075534	0.002658	4.48E-03
UBE2L3	22:21932068	rs181362	MVP	Intron	T/C	0.254	-0.007346	0.003075	1.69E-02
PEMT	17:17409560	rs7946	MVP	Nonsynonymous	T/C	0.6667	-0.00721	0.002916	1.34E-02

© 2020 Trinder M et al. *JAMA Cardiology.*

CDC25A	3:48229366	rs146179438	MVP	Nonsynonymous	A/C	0.01984	-0.007085	0.010004	4.79E-01
TTC39B	9:15305378	rs581080	MVP	Intron	C/G	0.7968	0.0068168	0.003349	4.18E-02
PCK1	20:56140439	rs41302559	MVP	Nonsynonymous	A/G	0.002059	-0.0067475	0.029184	8.17E-01
ZC3H4	19:47589895	rs2303108	MVP	Intron	C/T	0.6653	0.006731	0.002847	1.81E-02
NA	3:156798732	rs900399	MVP	Intergenic	G/A	0.3824	0.006631	0.002719	1.47E-02
NA	7:130433384	rs4731702	MVP	Intergenic	T/C	0.4596	-0.006628	0.002745	1.58E-02
NA	12:7691134	rs7136716	MVP	Intergenic	G/A	0.1502	-0.00607	0.003758	1.06E-01
NA	1:214159256	rs340874	MVP	Intergenic	C/T	0.5061	0.0059034	0.002671	2.71E-02
PINX1	8:10683929	rs11776767	MVP	Intron	C/G	0.3719	-0.0058878	0.002849	3.87E-02
PLCB3	11:64031241	rs35169799	MVP	Nonsynonymous	T/C	0.05893	0.005833	0.005616	2.99E-01
MMAB	12:110000193	rs7134594	MVP	Intron	T/C	0.5261	0.005685	0.002678	3.38E-02
NA	3:185834499	rs9816226	MVP	Intergenic	T/A	0.8257	0.005662	0.003499	1.06E-01
AKR1C4	10:5254847	rs1832007	MVP	Intron	G/A	0.1381	-0.0056006	0.003874	1.48E-01
F2	11:46743247	rs3136441	MVP	Intron	C/T	0.1514	0.0052327	0.003993	1.90E-01
NA	2:227100698	rs2972146	MVP	Intergenic	T/G	0.67	0.0052182	0.002901	7.21E-02
ZNF664|ZNF664-FAM101A	12:124460167	rs4765127	MVP	Intron	T/G	0.3294	-0.005202	0.004011	1.95E-01
MPP3	17:41878166	rs8077889	MVP	Downstream	C/A	0.2017	-0.005101	0.003288	1.21E-01
NA	6:139829666	rs605066	MVP	Intergenic	T/C	0.5692	-0.004976	0.002963	9.31E-02
ARL15	5:53298025	rs6450176	MVP	Intron	A/G	0.2703	0.004588	0.003222	1.54E-01
NA	13:51221618	rs797486	MVP	Intergenic	A/C	0.8709	0.0043901	0.003906	2.61E-01
FTO	16:53809247	rs1121980	MVP	Intron	A/G	0.4278	0.0040968	0.002687	1.27E-01
NA	18:56109859	rs8099014	MVP	Intergenic	A/C	0.7115	-0.0040945	0.002932	1.63E-01
ACP1	2:272203	rs11553746	MVP	Nonsynonymous	T/C	0.3288	0.003969	0.002834	1.61E-01
DNM3	1:172346548	rs1011731	MVP	Intron	A/G	0.5598	-0.003828	0.00273	1.61E-01
FAM13A	4:89740128	rs13133548	MVP	Intron	A/G	0.4835	0.003702	0.002635	1.60E-01
ATG7	3:11400249	rs2606736	MVP	Intron	T/C	0.599	-0.003689	0.002692	1.71E-01
NA	2:165540800	rs12328675	MVP	Intergenic	C/T	0.1197	-0.0036588	0.00406	3.67E-01
MET	7:116358044	rs38855	MVP	Intron	G/A	0.4624	-0.003534	0.002655	1.83E-01
NA	12:20473758	rs7134375	MVP	Intergenic	A/C	0.4116	0.0033477	0.002673	2.10E-01
STAB1	3:52532118	rs13326165	MVP	Intron	G/A	0.8052	0.0032941	0.003352	3.26E-01
LOC100507053	4:100014805	rs2602836	MVP	Intron	G/A	0.5796	0.003104	0.002781	2.64E-01

© 2020 Trinder M et al. *JAMA Cardiology.*

RSPO3	6:127452935	rs2745353	MVP	Intron	T/C	0.5253	0.0030394	0.002636	2.49E-01
CMIP	16:81534790	rs2925979	MVP	Intron	C/T	0.698	-0.002986	0.002856	2.96E-01
NA	5:67714246	rs4976033	MVP	Intergenic	G/A	0.4248	0.002944	0.002674	2.71E-01
STARD3	17:37813856	rs11869286	MVP	Intron	C/G	0.6304	0.0029173	0.002914	3.17E-01
FNIP1	5:131008194	rs26008	MVP	Nonsynonymous	C/T	0.9195	0.002729	0.00484	5.73E-01
CAPN3	15:42683787	rs2412710	MVP	Intron	A/G	0.0214	0.002442	0.009113	7.89E-01
RGS17	6:153431125	rs12055786	MVP	Intron	T/C	0.4369	-0.0023762	0.003009	4.30E-01
NA	18:57851097	rs17782313	MVP	Intergenic	C/T	0.2441	0.002343	0.003084	4.47E-01
NA	14:105277209	rs4983559	MVP	Intergenic	A/G	0.5689	-0.002315	0.002693	3.90E-01
INSR	19:7224431	rs7248104	MVP	Intron	A/G	0.403	-0.0022646	0.002681	3.98E-01
C1orf220	1:178515312	rs4650994	MVP	Intron	A/G	0.4916	0.002208	0.002681	4.10E-01
NA	11:75455021	rs499974	MVP	Intergenic	A/C	0.184	-0.002109	0.003449	5.41E-01
NBEAL2	3:47045846	rs2305637	MVP	Nonsynonymous	T/C	0.1581	0.0020226	0.004463	6.50E-01
PLA2G6	22:38546033	rs5756931	MVP	Intron	C/T	0.3691	-0.0019876	0.002905	4.94E-01
NA	8:121868551	rs4871137	MVP	Intergenic	T/G	0.6404	-0.001985	0.002862	4.88E-01
FUT8	14:65914867	rs10483776	MVP	Intron	G/A	0.1638	-0.0018306	0.003576	6.09E-01
PDE3B	11:14852490	rs1037378	MVP	Intron	A/G	0.5357	-0.0017962	0.002639	4.96E-01
FPR3	19:52324216	rs17695224	MVP	Intron	A/G	0.2508	-0.001773	0.003043	5.60E-01
RRNAD1	1:156700651	rs12145743	MVP	Intron	G/T	0.3121	-0.0016557	0.003117	5.95E-01
CTSB	8:11702375	rs3947	MVP	Utr3	A/G	0.2313	-0.0016426	0.003425	6.32E-01
NA	7:50305863	rs4917014	MVP	Intergenic	G/T	0.2985	-0.001572	0.002889	5.86E-01
NA	12:125261593	rs838880	MVP	Intergenic	T/C	0.6334	-0.0014224	0.002762	6.07E-01
CASP8	2:202122995	rs3769823	MVP	Nonsynonymous	G/A	0.6897	0.001299	0.002881	6.52E-01
PSKH1	16:67928042	rs16942887	MVP	Intron	A/G	0.1336	0.0012499	0.004045	7.57E-01
PEPD	19:33899065	rs731839	MVP	Intron	A/G	0.6336	0.001228	0.002756	6.56E-01
PABPC4	1:40028180	rs4660293	MVP	Intron	G/A	0.208	0.0009698	0.003299	7.69E-01
NA	10:115789375	rs7076938	MVP	Intergenic	T/C	0.7253	-0.0009664	0.00295	7.43E-01
FRMD5	15:44245931	rs2929282	MVP	Intron	T/A	0.06708	0.0009448	0.005531	8.64E-01
LINGO2	9:28414339	rs10968576	MVP	Intron	G/A	0.2891	-0.0008863	0.002918	7.61E-01
RIC8B	12:107174646	rs10861661	MVP	Intron	C/A	0.2332	0.0008568	0.00323	7.91E-01
DHX58	17:40257163	rs2074158	MVP	Nonsynonymous	C/T	0.1867	0.0008413	0.003924	8.30E-01

© 2020 Trinder M et al. *JAMA Cardiology.*

SLC25A30	13:45970147	rs138358301	MVP	Nonsynonymous	G/A	0.003473	0.0007869	0.022398	9.72E-01
ZC3H12C	11:109995944	rs746463	MVP	Intron	T/C	0.6892	0.0007307	0.002948	8.04E-01
NA	3:49971514	rs7613875	MVP	Intergenic	A/C	0.4941	0.0006262	0.002723	8.18E-01
BAHD1	15:40751555	rs3803357	MVP	Nonsynonymous	A/C	0.5429	0.0004181	0.002685	8.76E-01
IGHMBP2	11:68703959	rs622082	MVP	Nonsynonymous	G/A	0.3102	0.0003493	0.002873	9.03E-01
NA	19:54792761	rs386000	MVP	Intergenic	C/G	0.2189	-0.0002399	0.004104	9.53E-01
NA	15:58683366	rs1532085	MVP	Intergenic	G/A	0.5907	0.000173	0.00269	9.49E-01
GALNT2	1:230295691	rs4846914	MVP	Intron	A/G	0.5506	0.0001637	0.002833	9.54E-01

Data are derived from the Global Lipid Genetics Consortium (GLGC)[1]. This 223 SNV polygenic score for LDL-C has been published[2]. Variants are sorted in order of greatest to smallest effect size (beta) for their association with LDL-C levels, starting with the initial significant SNVs identified (Initial Description: GLGC2013)[1] and subsequently then SNVs included in the more recent LDL-C polygenic score[2]. Directionality of the reported effect size is based on the minor allele. Chr: chromosome; Pos: position; EA: effect allele; NEA: non-effect allele; EAF: estimated minor allele frequency; SE: standard error; Utr: untranslated region.

© 2020 Trinder M et al. *JAMA Cardiology.*

eTable 4. Baseline Characteristics of UK Biobank Participants at the Time of Study Enrollment

Characteristic	Genotyping Array Cohort (n=478,428)	Genotyping Array & Exome Sequencing (n=48,741)
**Demographics**		
Age - mean (SD)	56.6 (8.07)	56.64 (7.99)
Female sex - no. (%)	259400 (54.22)	26541 (54.45)
European ancestry - no. (%)	462236 (96.62)	46836 (96.09)
East Asian ancestry - no. (%)	11220 (2.35)	1294 (2.65)
African ancestry - no. (%)	4972 (1.04)	611 (1.25)
**Biochemistry - mean (SD) / n**		
Total cholesterol (mg/dL)	220.34 (44.25) / 456042	220.06 (43.99) / 45739
Direct LDL-C (mg/dL)	141.03 (34.39) / 455191	140.33 (34.14) / 45666
Apolipoprotein B (mg/dL)	103.28 (23.81) / 453762	102.93 (23.56) / 45528
Triglycerides (mg/dL)	155.12 (91.04) / 455681	150.82 (87.53) / 45690
HDL-C (mg/dL)	56.03 (14.79) / 417368	56.98 (14.94) / 43048
Apolipoprotein A1 (mg/dL)	153.85 (27.06) / 415065	155.51 (27.19) / 42752
Lipoprotein(a) (mg/dL)	18.48 (20.49) / 364458	18.46 (20.51) / 36575
Hemoglobin A1c (%)	5.45 (0.61) / 455135	5.44 (0.58) / 45493
C-reactive protein (mg/dL)	0.26 (0.44) / 455044	0.25 (0.43) / 45632
**Physical Exam - mean (SD) / n**		
Body mass index (kg/m²)	27.41 (4.78) / 476579	27.40 (4.80) / 48584
**Medical History - no. (%) / n**		
Angina	15384 (3.23) / 476846	1427 (2.93) / 48666
Myocardial infarction	11085 (2.32) / 476846	1016 (2.09) / 48666
Stroke	7256 (1.52) / 476846	672 (0.14) / 48666
Hypertension	128493 (26.95) / 476846	12759 (26.22) / 48666
Diabetes	24524 (5.15) / 476511	2476 (5.09) / 48624
Current smoker	50111 (10.53) / 476066	4363 (8.98) / 48582
**Medications - no. (%) / n**		
Cholesterol-lowering medication	32934 (12.78) / 257611	3366 (12.75) / 26402
Antihypertensives	45260 (17.57) / 257611	4473 (16.94) / 26402
Insulin	2089 (0.81) / 257611	208 (0.79) / 26402
Exogenous hormones	25416 (9.87) / 257611	2651 (10.04) / 26402

HDL-C: high-density lipoprotein cholesterol; LDL-C: low-density lipoprotein cholesterol; SD: standard deviation.

© 2020 Trinder M et al. *JAMA Cardiology.*

eTable 5. FH-causing Variants Identified in the *LDLR*, *APOB*, and *PCSK9* Genes

Gene	Chr:Pos	Ref/Alt	rsID	Variant	ClinVar Significance	n
LDLR	19:11129602	G/A	rs137853964	missense_variant	Conflicting_interpretations_of_pathogenicity	16
LDLR	19:11111538	A/C	rs138315511	missense_variant	Conflicting_interpretations_of_pathogenicity	15
LDLR	19:11105414	G/A	rs139089530	missense_variant	Uncertain_significance	11
LDLR	19:11110660	G/A	rs746834464	missense_variant	Pathogenic/Likely_pathogenic	11
LDLR	19:11107506	A/C	rs761765254	missense_variant	Likely_pathogenic	7
LDLR	19:11129564	G/A	rs5928	missense_variant	Conflicting_interpretations_of_pathogenicity	7
LDLR	19:11100340	C/T	rs376207800	missense_variant	Conflicting_interpretations_of_pathogenicity	6
LDLR	19:11102774	G/A	rs144172724	missense_variant	Pathogenic/Likely_pathogenic	6
LDLR	19:11120436	C/T	rs28942084	missense_variant	Pathogenic/Likely_pathogenic	6
LDLR	19:11120501	G/A	rs879255142	missense_variant	Likely_pathogenic	6
LDLR	19:11113620	G/A	rs139624145	missense_variant	Conflicting_interpretations_of_pathogenicity	5
LDLR	19:11120483	G/A	rs368838866	missense_variant	Conflicting_interpretations_of_pathogenicity	5
LDLR	19:11120488	G/A	rs140731590	missense_variant	Conflicting_interpretations_of_pathogenicity	5
LDLR	19:11116141	G/A	rs759876319	missense_variant	Pathogenic/Likely_pathogenic	4
LDLR	19:11116970	C/A	rs1410295777	missense_variant	Pathogenic	4
LDLR	19:11120157	C/T	rs373570349	stop_gained	Conflicting_interpretations_of_pathogenicity	4
LDLR	19:11129633	A/G	rs150631538	missense_variant		4
LDLR	19:11131384	AAGAC/A	rs773717642	frameshift_variant		4
LDLR	19:11100237	G/A	rs551747280	missense_variant	Likely_pathogenic	3
LDLR	19:11102705	C/T	rs370860696	missense_variant	Likely_pathogenic	3
LDLR	19:11102765	G/A	rs750474121	missense_variant	Likely_pathogenic	3
LDLR	19:11105408	G/A	rs200727689	missense_variant	Pathogenic/Likely_pathogenic	3
LDLR	19:11105568	A/G	rs373822756	missense_variant	Pathogenic/Likely_pathogenic	3
LDLR	19:11110759	C/T	rs769737896	stop_gained	Pathogenic	3
LDLR	19:11116873	C/T	rs185098634	missense_variant	Conflicting_interpretations_of_pathogenicity	3
LDLR	19:11120114	T/C	rs141155833	missense_variant	Likely_pathogenic	3
LDLR	19:11120212	C/A	rs762815611	missense_variant	Conflicting_interpretations_of_pathogenicity	3
LDLR	19:11120454	C/T	rs369943481	missense_variant	Uncertain_significance	3
LDLR	19:11120480	G/A	rs375009082	missense_variant	Uncertain_significance	3

© 2020 Trinder M et al. *JAMA Cardiology.*

LDLR	19:11129510	C/T	rs755033580	stop_gained		3
LDLR	19:11102732	T/G	rs121908025	missense_variant	Pathogenic/Likely_pathogenic	2
LDLR	19:11102739	G/A	rs875989894	missense_variant	Pathogenic/Likely_pathogenic	2
LDLR	19:11105556	ATGG/A	rs121908027	disruptive_inframe_deletion	Pathogenic/Likely_pathogenic	2
LDLR	19:11105585	GAC/G	rs387906305	frameshift_variant	Pathogenic/Likely_pathogenic	2
LDLR	19:11106588	G/A	rs768563000	missense_variant	Conflicting_interpretations_of_pathogenicity	2
LDLR	19:11110714	G/A	rs544453230	missense_variant	Conflicting_interpretations_of_pathogenicity	2
LDLR	19:11113329	C/T	rs368562025	missense_variant	Conflicting_interpretations_of_pathogenicity	2
LDLR	19:11113705	C/T	rs755154048	missense_variant	Likely_pathogenic	2
LDLR	19:11113751	T/G	rs763884010	missense_variant		2
LDLR	19:11116095	T/G	rs875989924	missense_variant&<br>splice_region_variant	Uncertain_significance	2
LDLR	19:11116198	A/G	rs758194385	missense_variant	Likely_pathogenic	2
LDLR	19:11120143	C/T	rs746118995	missense_variant	Pathogenic/Likely_pathogenic	2
LDLR	19:11123315	C/T	rs138477254	missense_variant	Conflicting_interpretations_of_pathogenicity	2
LDLR	19:11129571	G/C	rs1399689294	missense_variant	Conflicting_interpretations_of_pathogenicity	2
LDLR	19:11129669	C/T	rs377437226	stop_gained&<br>splice_region_variant	Uncertain_significance	2
LDLR	19:11100272	CA/C	rs879254413	frameshift_variant	Pathogenic	1
LDLR	19:11100294	G/A	rs778284147	missense_variant	Conflicting_interpretations_of_pathogenicity	1
LDLR	19:11100328	A/T		missense_variant		1
LDLR	19:11102683	CG/C	rs1412805126	frameshift_variant	Pathogenic	1
LDLR	19:11102699	G/T	rs574337291	missense_variant	Conflicting_interpretations_of_pathogenicity	1
LDLR	19:11102714	C/T	rs730882078	missense_variant	Conflicting_interpretations_of_pathogenicity	1
LDLR	19:11102785	TCG/T	rs875989896	frameshift_variant&<br>splice_donor_variant&<br>splice_region_variant&<br>intron_variant	Pathogenic	1
LDLR	19:11102787	G/C	rs112029328	splice_donor_variant&<br>intron_variant	Pathogenic/Likely_pathogenic	1
LDLR	19:11105249	C/T	rs774723292	missense_variant	Conflicting_interpretations_of_pathogenicity	1
LDLR	19:11105407	C/A	rs752596535	stop_gained	Pathogenic/Likely_pathogenic	1
LDLR	19:11105415	A/G	rs533896621	missense_variant		1
LDLR	19:11105526	G/A	rs759737884	missense_variant		1
LDLR	19:11105587	C/G	rs121908028	missense_variant	Pathogenic/Likely_pathogenic	1

© 2020 Trinder M et al. *JAMA Cardiology.*

LDLR	19:11105588	G/T	rs121908029	stop_gained	Pathogenic/Likely_pathogenic	1
LDLR	19:11106592	T/C	rs748131256	missense_variant	Likely_pathogenic	1
LDLR	19:11106593	C/A		missense_variant		1
LDLR	19:11106601	C/G	rs1208667598	missense_variant	Pathogenic	1
LDLR	19:11106639	C/T	rs200990725	missense_variant	Conflicting_interpretations_of_pathogenicity	1
LDLR	19:11106668	T/A	rs139043155	missense_variant	Conflicting_interpretations_of_pathogenicity	1
LDLR	19:11106685	A/C	rs202213054	missense_variant& splice_region_variant	Uncertain_significance	1
LDLR	19:11107433	G/A	rs375495026	missense_variant	Likely_pathogenic	1
LDLR	19:11107440	G/C		missense_variant		1
LDLR	19:11107461	G/A	rs879254707	missense_variant	Likely_pathogenic	1
LDLR	19:11107481	C/T	rs151207122	missense_variant	Conflicting_interpretations_of_pathogenicity	1
LDLR	19:11107486	C/G	rs875989909	missense_variant	Pathogenic/Likely_pathogenic	1
LDLR	19:11110690	C/T	rs747507019	missense_variant	Conflicting_interpretations_of_pathogenicity	1
LDLR	19:11111514	A/G	rs755449669	missense_variant& splice_region_variant	Likely_pathogenic	1
LDLR	19:11111609	G/T	rs1402951356	missense_variant	Uncertain_significance	1
LDLR	19:11111619	C/T	rs149227308	missense_variant	Conflicting_interpretations_of_pathogenicity	1
LDLR	19:11113287	C/A	rs875989918	missense_variant	Likely_pathogenic	1
LDLR	19:11113307	C/T	rs121908043	missense_variant	Pathogenic/Likely_pathogenic	1
LDLR	19:11113308	G/A	rs552422789	missense_variant	Conflicting_interpretations_of_pathogenicity	1
LDLR	19:11113313	G/A	rs137943601	missense_variant	Conflicting_interpretations_of_pathogenicity	1
LDLR	19:11113343	G/A	rs869320651	missense_variant	Likely_pathogenic	1
LDLR	19:11113376	G/A	rs28942078	missense_variant	Pathogenic/Likely_pathogenic	1
LDLR	19:11113382	G/C	rs28942079	missense_variant	Likely_pathogenic	1
LDLR	19:11113650	G/A	rs373646964	missense_variant	Conflicting_interpretations_of_pathogenicity	1
LDLR	19:11113678	C/T	rs755667663	missense_variant	Pathogenic/Likely_pathogenic	1
LDLR	19:11113684	C/A	rs967205800	missense_variant		1
LDLR	19:11113743	G/A	rs28942080	missense_variant	Pathogenic/Likely_pathogenic	1
LDLR	19:11116099	T/A	rs779913921	missense_variant		1
LDLR	19:11116132	T/A		missense_variant		1
LDLR	19:11116144	G/T	rs28942081	missense_variant	Likely_pathogenic	1
LDLR	19:11116898	T/C	rs875989930	missense_variant	Likely_pathogenic	1

© 2020 Trinder M et al. *JAMA Cardiology.*

LDLR	19:11116900	C/T	rs730882109	missense_variant	Conflicting_interpretations_of_pathogenicity	1
LDLR	19:11116925	ACGG/A	rs1221971156	disruptive_inframe_deletion		1
LDLR	19:11116936	C/T	rs373371572	missense_variant	Conflicting_interpretations_of_pathogenicity	1
LDLR	19:11116988	C/T	rs377449975	missense_variant	Pathogenic	1
LDLR	19:11120094	C/T		stop_gained&splice_region_variant		1
LDLR	19:11120106	G/T	rs875989933	missense_variant	Likely_pathogenic	1
LDLR	19:11120143	C/A		missense_variant		1
LDLR	19:11120144	G/A	rs754536745	missense_variant	Conflicting_interpretations_of_pathogenicity	1
LDLR	19:11120370	G/A	rs879255102	missense_variant&splice_region_variant	Likely_pathogenic	1
LDLR	19:11120408	G/A	rs745753810	missense_variant	Conflicting_interpretations_of_pathogenicity	1
LDLR	19:11120411	T/C	rs775092314	missense_variant	Pathogenic/Likely_pathogenic	1
LDLR	19:11120419	T/A	rs760436036	stop_gained	Pathogenic	1
LDLR	19:11120424	G/C	rs201637900	missense_variant	Likely_pathogenic	1
LDLR	19:11120441	A/T	rs1060499932	missense_variant	Uncertain_significance	1
LDLR	19:11120442	T/TC	rs137853965	frameshift_variant	Pathogenic	1
LDLR	19:11120484	G/T		missense_variant		1
LDLR	19:11120488	G/C	rs140731590	missense_variant		1
LDLR	19:11129582	G/A		missense_variant		1
LDLR	19:11129602	G/T	rs137853964	missense_variant	Likely_pathogenic	1
LDLR	19:11129609	A/T		stop_gained		1
LDLR	19:11129623	G/A		missense_variant		1
APOB	2:21038086	C/A	rs766243954	stop_gained	Pathogenic	3
APOB	2:21006129	T/C	rs762028704	missense_variant	Pathogenic	2
APOB	2:21002392	CAT/C	rs760832994	frameshift_variant	Pathogenic	1
APOB	2:21006128	G/C	rs150312765	missense_variant	Pathogenic	1
PCSK9	1:55043921	C/T	rs185392267	missense_variant	Pathogenic	3
PCSK9	1:55058543	C/G	rs772677312	structural_interaction_variant	Likely_pathogenic	3
PCSK9	1:55039995	C/G	rs11583680	missense_variant	Pathogenic	2
PCSK9	1:55058570	C/T	rs761767572	structural_interaction_variant	Pathogenic	2
PCSK9	1:55052364	G/A	rs793888521	missense_variant	Likely_pathogenic	1
PCSK9	1:55052650	G/A	rs768795323	missense_variant&splice_region_variant	Pathogenic	1

© 2020 Trinder M et al. *JAMA Cardiology.*

PCSK9	1:55057454	G/T	rs137852912	structural_interaction_variant	Pathogenic/Likely_pathogenic	1

Chr: chromosome; Pos: position; Ref/Alt: reference/alternative.

© 2020 Trinder M et al. *JAMA Cardiology.*

eTable 6. Baseline Characteristics of UK Biobank Participants in the Exome Sequencing Cohort Stratified by Monogenic FH

Characteristic	No monogenic FH (n=48,464)	Monogenic FH (n=277)	p
**Demographics**			
Age - mean (SD)	56.64 (7.99)	57.08 (7.87)	0.39
Female sex - no. (%)	26384 (54.44)	157 (56.68)	0.49
European ancestry - no. (%)	46576 (96.10)	260 (93.86)	0.08
East Asian ancestry - no. (%)	1283 (2.65)	11 (3.97)	0.24
African ancestry - no. (%)	605 (1.25)	6 (2.17)	0.27
**Biochemistry - mean (SD) / n**			
Total cholesterol (mg/dL)	219.99 (43.89) / 45482	232.91 (57.43) / 257	<0.001
Direct LDL-C (mg/dL)	140.21 (34.00) / 45410	161.15 (49.14) / 256	<0.001
Apolipoprotein B (mg/dL)	102.88 (23.54) / 45275	110.86 (26.78) / 253	<0.001
Triglycerides (mg/dL)	150.91 (87.59) / 45435	134.55 (75.22) / 255	<0.001
HDL-C (mg/dL)	56.97 (14.94) / 42804	57.4 (14.58) / 244	0.52
Apolipoprotein A1 (mg/dL)	155.52 (27.19) / 42508	152.83 (25.8) / 244	0.29
Lipoprotein(a) (mg/dL)	18.46 (20.52) / 36381	18.86 (18.74) / 194	0.12
Hemoglobin A1c (%)	5.44 (0.58) / 45236	5.48 (0.56) / 257	0.06
C-reactive protein (mg/dL)	0.25 (0.43) / 45377	0.25 (0.44) / 255	0.05
**Physical Exam - mean (SD) / n**			
Body mass index (kg/m²)	27.40 (4.80) / 48308	27.59 (4.97) / 276	0.56
**Medical History - no. (%) / n**			
Angina	1411 (2.92) / 48391	16 (5.82) / 275	0.008
Myocardial infarction	999 (2.06) / 48391	17 (6.18) / 275	<0.001
Stroke	669 (1.38) / 48391	3 (0.01) / 275	0.88
Hypertension	12686 (26.22) / 48391	73 (26.55) / 275	0.96
Diabetes	2460 (5.09) / 48347	16 (5.78) / 277	0.70
Current smoker	4347 (9.00) / 48305	16 (5.78) / 277	0.08
**Medications - no. (%) / n**			
Cholesterol-lowering medication	3309 (12.61) / 26245	57 (36.31) / 157	<0.001
Antihypertensives	4439 (16.91) / 26245	34 (21.66) / 157	0.14
Insulin	208 (0.79) / 26245	0 (0) / 157	0.50
Exogenous hormones	2643 (10.07) / 26245	8 (5.10) / 157	0.05

HDL-C: high-density lipoprotein cholesterol; ; LDL-C: low-density lipoprotein cholesterol; SD: standard deviation.

© 2020 Trinder M et al. *JAMA Cardiology.*

eFigure 1. Classification of Patients by Genetic Ancestry Superpopulation

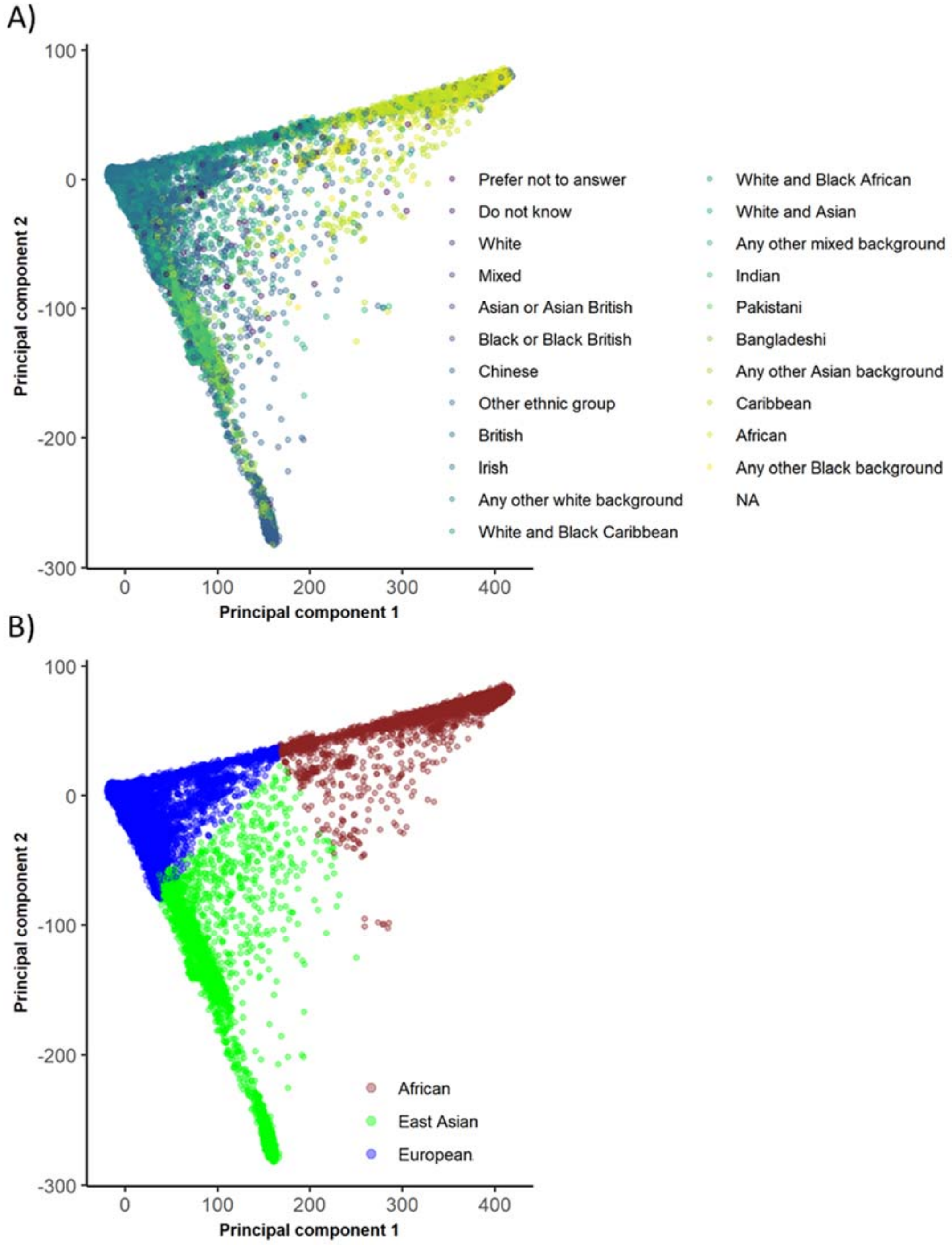

(A) The first 2 principal components of genetic ancestry for individuals from the UK Biobank genotyping array cohort are displayed and colored by (A) self-reported ancestry and (B) clustering of individuals into African, East Asian, and European superpopulations.

eFigure 2. Percent Variance in LDL-C Levels Explained by Single-Nucleotide Variants (SNVs) in the LDL-C Polygenic Score

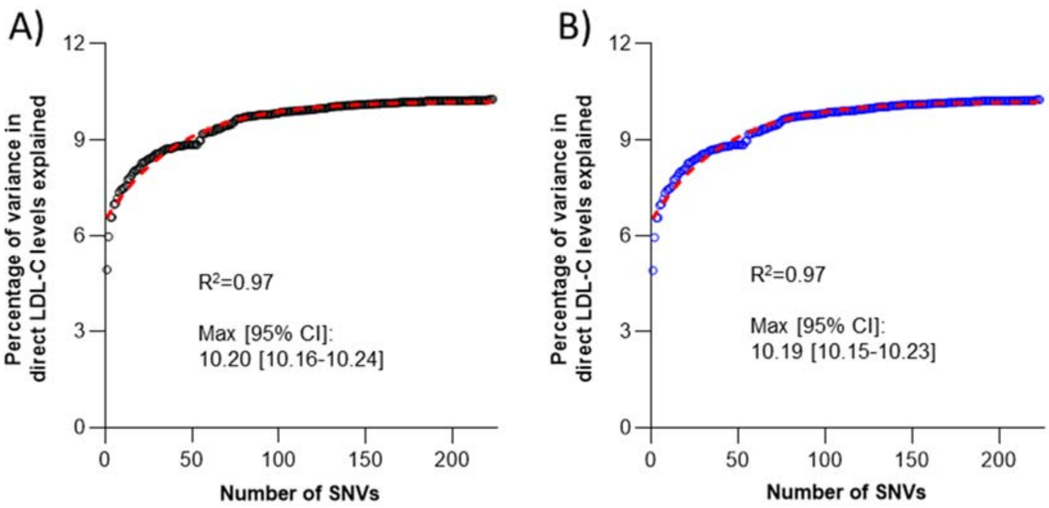

The percent variance of LDL-C levels explained by the LDL-C polygenic score is displayed for the stepwise addition of the 223 SNVs into multivariable linear regression models with LDL-C levels as the dependent variable. Linear regression models were adjusted for age, sex, the first 4 principal components of genetic ancestry, and genotyping array and batch and included (A) all individuals (n= 239,826) and (B) individuals of European genetic ancestry (238,058) with no missing SNVs from the 223 SNV LDL-C polygenic score. SNVs were added in the order displayed in Online-Only Table 2. The red dashed line depicts the exponential plateau model of the data.

© 2020 Trinder M et al. *JAMA Cardiology.*

eFigure 3. Measured Levels of LDL-C Versus Decile of LDL-C Polygenic Score Percentile

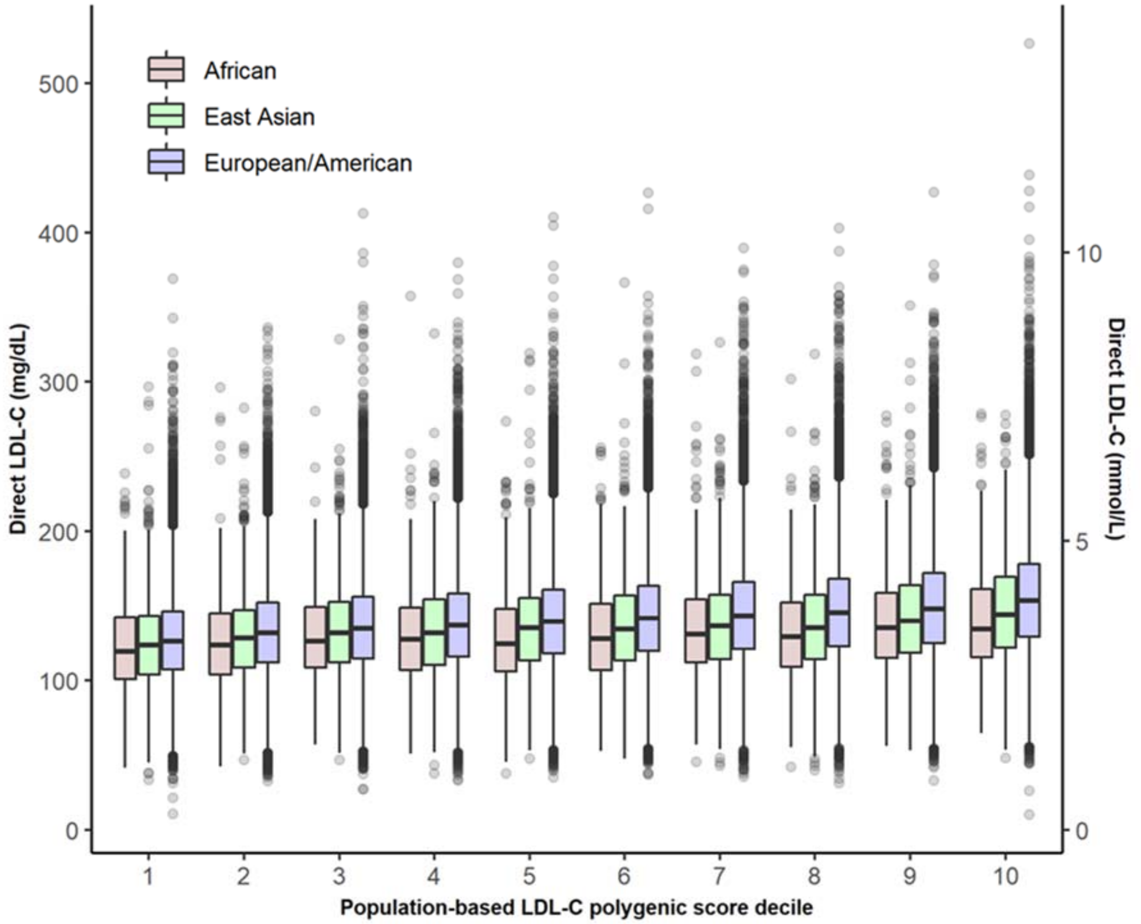

Boxplots display the median, interquartile range, and minimum to maximum of direct LDL-C levels versus the superpopulation-specific LDL-C polygenic score percentile.

© 2020 Trinder M et al. *JAMA Cardiology.*

eFigure 4. LDL-C Polygenic Scores are Associated With Risk of CVD Among the East Asian and European Superpopulations

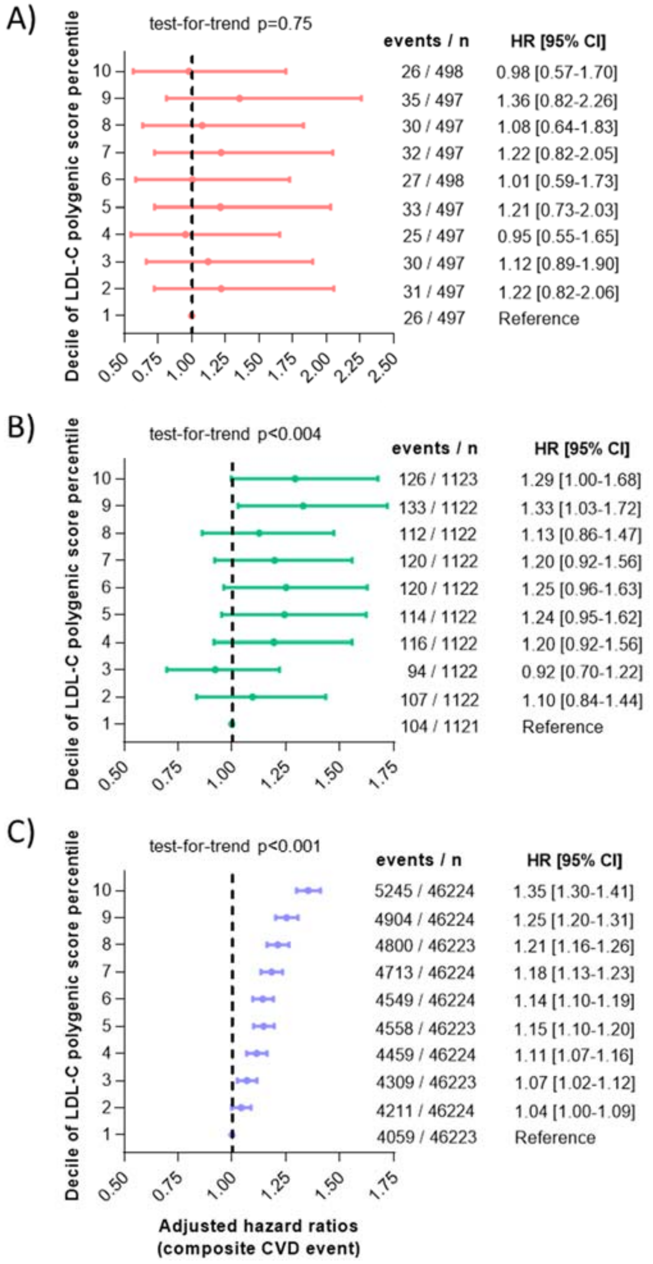

The adjusted hazard ratios ± 95% confidence intervals for composite CVD events of myocardial infarction, coronary or carotid revascularization, stroke, or all-cause mortality are displayed for each decile of LDL-C polygenic score percentile are stratified by (A) African, (B) East Asian, and (C) Europeans superpopulations. Hazard ratios were adjusted for age, sex, genotyping array/batch, and the first 4 principal components of ancestry.

© 2020 Trinder M et al. *JAMA Cardiology.*

eFigure 5. Impact of ClinVar Pathogenic Versus Predicted FH-causing Variants on Premature CVD Events

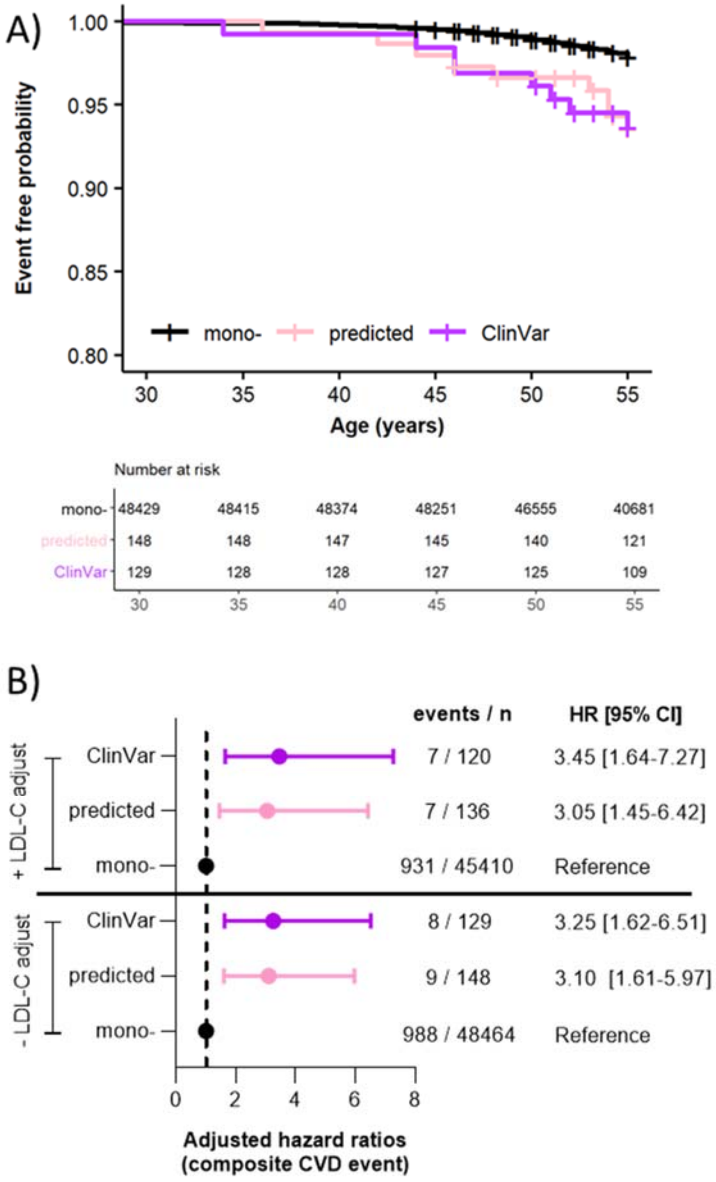

(A) Time-to-first-event analyses are displayed for premature CVD events (experienced at age 55 years-of-age or younger), including myocardial infarction, coronary or carotid revascularization, stroke, or all-cause mortality. Individuals were stratified based on the rank order groupings of FH-causing variants classified as ‘pathogenic’ or ‘likely pathogenic’ in ClinVar, predicted FH-causing variants, and no identified FH-causing variants. (B) Hazard ratios ± 95% confidence intervals are displayed for composite CVD events with or without adjustment for LDL-C levels. All hazard ratios were adjusted for age, sex, genotyping array/batch, and the first 4 principal components of ancestry.

© 2020 Trinder M et al. *JAMA Cardiology.*

eFigure 6. Impact of Monogenic FH-causing Variants on CVD Events

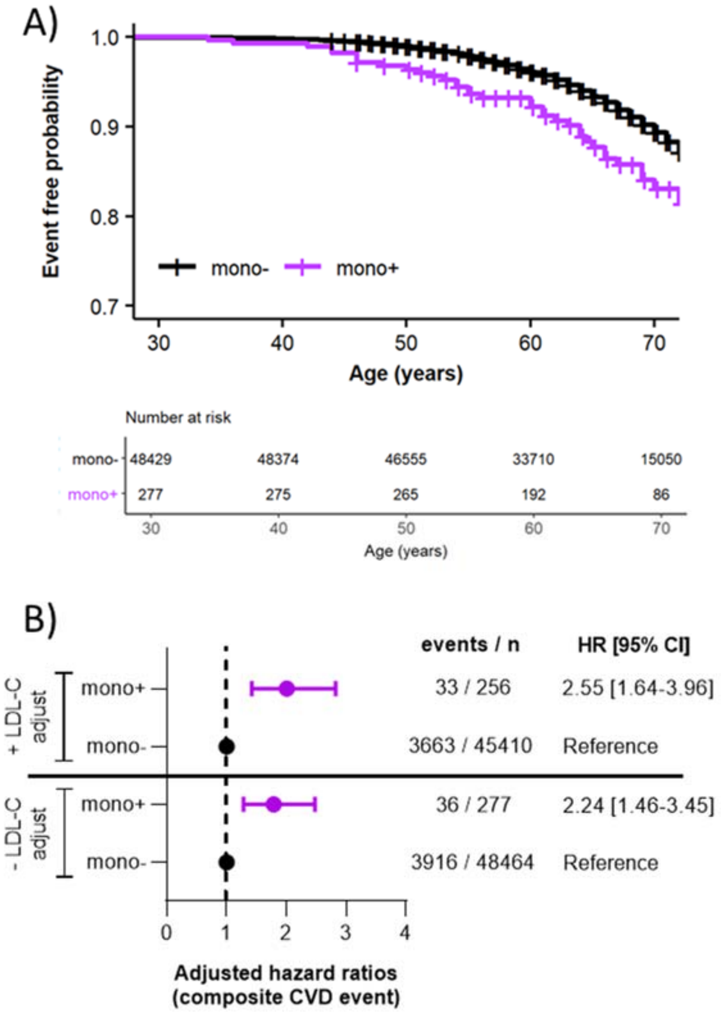

(A) Time-to-first-event analyses are displayed for composite CVD events, including myocardial infarction, coronary or carotid revascularization, stroke, or all-cause mortality. Individuals were stratified based on those that carried a monogenic FH-causing variant versus those that did not carry a monogenic FH-causing variant. (B) Hazard ratios ± 95% confidence intervals are displayed for composite CVD events with or without adjustment for LDL-C levels. All hazard ratios were adjusted for age, sex, genotyping array/batch, and the first 4 principal components of ancestry.

© 2020 Trinder M et al. *JAMA Cardiology.*

eFigure 7. Hypercholesterolemia is Underdiagnosed and Undertreated

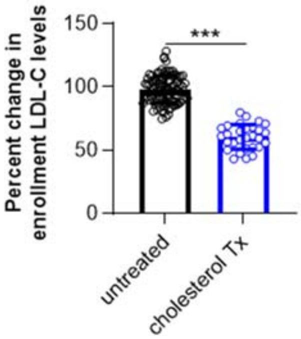

There were 128 individuals with LDL-C levels ≥ 193.35 mg/dL (5 mmol/L) that were not receiving cholesterol-lowering medication at enrollment and had follow-up data available. At follow-up, 29 of these individuals reported recceing cholesterol-lowering medication (cholesterol Tx) and 99 of these individuals reported that they were not taking cholesterol-lowering medication (untreated). The intraindividual percent change in follow-up LDL-C levels are depicted relative to their levels measured at enrollment. Data are displayed as means ± SD. Tx: treatment with cholesterol-lowering medication. ***$p<0.001$.

© 2020 Trinder M et al. *JAMA Cardiology.*

eReferences

1. Willer CJ, Schmidt EM, Sengupta S, et al; Global Lipids Genetics Consortium. Discovery and refinement of loci associated with lipid levels. *Nat Genet*. 2013;45(11):1274-1283. Medline:24097068 doi:10.1038/ng.2797

2. Klarin D, Damrauer SM, Cho K, et al; Global Lipids Genetics Consortium; Myocardial Infarction Genetics 18 (MIGen) Consortium; Geisinger-Regeneron DiscovEHR Collaboration; VA Million Veteran Program. Genetics of blood lipids among ~300,000 multi-ethnic participants of the Million Veteran Program. *Nat Genet*. 2018;50(11):1514-1523. Medline:30275531 doi:10.1038/s41588-018-0222-9

© 2020 Trinder M et al. *JAMA Cardiology.*
